# Addressing sufficiency of the CB1 receptor for endocannabinoid-mediated functions through conditional genetic rescue in forebrain GABAergic neurons

**DOI:** 10.1007/s00429-017-1411-5

**Published:** 2017-04-09

**Authors:** Floortje Remmers, Maren D. Lange, Martina Hamann, Sabine Ruehle, Hans-Christian Pape, Beat Lutz

**Affiliations:** 1 Institute of Physiological Chemistry, University Medical Center of the Johannes Gutenberg University Mainz, 55128 Mainz, Germany; 2 Institute of Physiology I, Westfaelische Wilhelms-University, 48149 Muenster, Germany

**Keywords:** Endocannabinoid, GABA, Seizure susceptibility, Anxiety, Fear extinction

## Abstract

Genetic inactivation of the cannabinoid CB1 receptor gene in different cell types in the brain has previously revealed necessary functions for distinct synaptic plasticity processes and behaviors. Here, we sought to identify CB1 receptor expression sites that are minimally required to reconstruct normal phenotypes. In a CB1-null background, we re-expressed endogenous CB1 receptors in forebrain GABAergic neurons, thereby assessing the sufficiency of CB1 receptors. Depolarization-induced suppression of inhibitory, but not excitatory, transmission was restored in hippocampal and amygdalar circuits. GABAergic CB1 receptors did not convey protection against chemically induced seizures, but prevented the spontaneous mortality observed in CB1 null mutants. Rescue of GABAergic CB1 receptors largely restored normal anxiety-like behavior but improved extinction of learned fear only marginally. This study illustrates that the approach of genetic reconstruction of complex behaviors is feasible. It also revealed distinct degrees of modulation for different emotional behaviors by the GABAergic population of CB1 receptors.

**Electronic supplementary material:**

The online version of this article (doi:10.1007/s00429-017-1411-5) contains supplementary material, which is available to authorized users.

## Introduction

In the brain, the cannabinoid type 1 (CB1) receptor is the major conveyor of cannabinoid signaling (Kano et al. 2009; Katona and Freund 2012). Retrograde activation of this mostly presynaptic receptor results in suppression of neurotransmission and hence in endocannabinoid-mediated synaptic plasticity. The endocannabinoid system serves many functions and is involved in the regulation of numerous processes. These include seizure susceptibility (e.g., Soltesz et al. 2015) and both innate and learned fear or anxiety (e.g., Ruehle et al. 2012; Aliczki and Haller 2015; Metna-Laurent et al. 2015; Lutz et al. 2015). Considering that the CB1 receptor is expressed, among others, in both glutamatergic and GABAergic neurons (Marsicano and Lutz 1999), many efforts have been made to dissect the specific involvement of the receptor in these two neuronal subpopulations in different endocannabinoid functions. Conditional knockout mice for the CB1 receptor have proven useful to assess the necessity of the receptor present in certain neuronal subpopulations (e.g., Monory et al. 2006; Metna-Laurent et al. 2012; Dubreucq et al. 2012; Rey et al. 2012; Llorente-Berzal et al. 2015). With this strategy, a conditional knockout of a subpopulation of the receptor that is necessary results in a “breakdown” of the normal behavior to the phenotype seen in CB1 null-mutants, whereas sufficiency of subpopulations can be assessed by investigating which subpopulations are required to “reconstruct” the normal behavior. Therefore, more recently we and others have begun to delineate subpopulations of the receptor that are sufficient for endocannabinoid functions with the conditional rescue mouse for the CB1 receptor (Ruehle et al. 2013; Soria-Gómez et al. 2014; de Salas-Quiroga et al. 2015; Lange et al. 2017). The reported sufficiency of the dorsal telencephalic glutamatergic CB1 receptor population has mostly matched its necessity, with the major exception of freezing during cued fear extinction (Ruehle et al. 2013). Although glutamatergic CB1 receptor was previously found to be necessary for appropriate fear extinction (Metna-Laurent et al. 2012; Dubreucq et al. 2012; Llorente-Berzal et al. 2015), no sufficiency was observed (Ruehle et al. 2013).

Here, we use a strategy for cell-type-specific CB1 receptor rescue (Ruehle et al. 2013), to obtain animals with endogenous levels of CB1 expression solely in forebrain GABAergic neurons in an otherwise functional knockout mouse. After verification of the efficacy and specificity of the CB1-rescue in forebrain GABAergic neurons (GABA-CB1-RS), we assessed the sufficiency of the GABAergic CB1 receptor subpopulation for several endocannabinoid functions, with a focus on aversive emotional processing.

## Methods

### Experimental animals

Adult male mice (2–6 months) were housed with littermates [from weaning, 1–5 animals per cage (average 3.1 for all genotypes in behavioral batches) in standard Makrolon type II cages, 267 × 207 × 140 mm] in a temperature- and humidity-controlled room (22.5 ± 1 °C; 60 ± 1%) with a 12–12-h light–dark cycle and access to food and water ad libitum. All experiments were carried out in accordance with the European Community’s Council Directive of 22 September 2010 (2010/63EU) and approved by the local animal care committee (Mainz: 23 177-07/G 13-1-021 and 23 177-07/A 13-1-003; Muenster: LANUV-NRW 8.87-51.05.20.10.218 and 84-02.05.50.15.032). All animals used in this study were bred in-house and were allowed to acclimatize to the behavioral unit for at least 1 week. In the Stop-CB1 mouse line, the CB1 receptor is silenced by a loxP flanked stop-cassette (an SV40 promoter-driven neomycin-resistance coding sequence, a HSV-TK polyadenylation sequence and two additional AATAAA sequences) in its 5′ UTR and can be conditionally activated by Cre recombinase under the control of its endogenous promoter and regulatory elements (Ruehle et al. 2013). In the present study, the CB1 receptor was rescued either globally (CB1-RS) or in forebrain GABAergic neurons (GABA-CB1-RS). Mice used for the experiments came from different mouse lines: CB1-KO (Marsicano et al. 2002), GABA-CB1-RS mice and their Stop-CB1 littermates, and wildtype-like CB1-RS mice. CB1-RS mice were generated as reported previously (Ruehle et al. 2013), by crossing Stop-CB1 mice with EIIA-Cre mice (Lakso et al. 1996). GABA-CB1-RS mice were generated by crossing Stop-CB1 mice with Dlx5/6-Cre mice (Zerucha et al. 2000; Monory et al. 2006). To avoid excision of the stop cassette in the female germ line (Massa et al. 2010), the Dlx5/6-Cre-positive parent in the GABA-CB1-RS mouse line was always male. All mouse lines used were backcrossed to C57BL/6J background for at least seven generations. Primers for genotyping of the different CB1-rescue mouse lines are given in supplementary Table S1. All mice were genotyped both before and at the conclusion of experiments.

### Receptor autoradiography

Adult male mice (3–6 months) were decapitated under isoflurane anesthesia (Forene, B506, Abbott GmbH & Co. KG, Wiesbaden, Germany), and brains were isolated and stored at −80 °C. Autoradiography was performed as previously described (Herkenham et al. 1991; Ruehle et al. 2013) on mounted 20-µm thick coronal cryosections. Brain sections were thawed, then incubated for 3 h at 30 °C in 50 mM Tris–HCl buffer (pH 7.4) containing 5% (w/v) fatty acid-free BSA (Carl Roth GmbH + Co. KG, Karlsruhe, Germany) with the addition of 5 nM ^3^H-CP 55,940 (specific activity 124 Ci/mmol; PerkinElmer, Germany). Non-specific binding was determined by incubating adjacent sections in 5 nM ^3^H-CP 55,940 in the presence of 10 µM cold CP 55,940 (Tocris Bioscience, Bristol, UK). After incubation, sections were washed twice for 90 min at 4 °C in 50 mM Tris–HCl buffer (pH 7.4) containing 1% BSA and briefly dipped in distilled water and dried overnight. TR tritium phosphor screens (PerkinElmer) were exposed to the slides together with a tritium standard (American Radiolabeled Chemicals, St. Louis, MO, USA) for 65 h and then scanned with a cyclone plus storage phosphor system (PerkinElmer). Ligand binding to the CB1 receptor was quantified using Optiquant software (PerkinElmer). A standard curve was compiled using the tritium standard. Brain regions were identified by comparison with the mouse brain atlas (Franklin and Paxinos 2007) and outlined manually for determination of average density. Right and left sides were pooled from four (globus pallidus) to ten (hippocampus) sections containing each region. Unspecific binding was subtracted, and the average density was calculated for each animal. Individual densities were expressed relative to the mean CB1-RS value and then averaged per genotype. The relative values found for GABA-CB1-RS were compared to those previously reported for Glu-CB1-RS (Ruehle et al. 2013) to obtain an estimation of the relative contribution of the receptor in both neuronal populations in wildtype mice. For this purpose, the previously reported values were now expressed relative to those of CB1-RS rather than to wildtype mice and two regions that were originally not measured for Glu-CB1-RS animals and their (Stop-CB1 and CB1-RS) controls (entopeduncular nucleus and substantia nigra) were now also quantified from the original scans for this purpose (supplementary Fig. S1).

### Immunohistochemistry

Adult male mice (2.5–4 months) were transcardially perfused with phosphate-buffered saline (PBS) and then 4% paraformaldehyde (PFA; Roti-Histofix 4%, Carl Roth) in PBS under deep pentobarbital anesthesia supported by buprenorphine analgesia. Brains were isolated and postfixed for 24 h in 4% PFA, treated with 30% sucrose/PBS solution for 48 h, and then stored at −80 °C until sectioning. Coronal sections (40 µm thick) were prepared on a cryostat and stored in cryoprotection solution (25% glycerin, 25% ethylene glycol, 50% PBS) until use. Immunostaining was performed on free-floating sections containing the hippocampus or amygdala. After a 5-min wash in 0.2% Triton X-100 in PBS (PBS-TX) and blocking in 4% goat serum in PBS-TX for 15 min, sections were incubated at 4 °C overnight with primary antibody (rabbit anti-CB1, 1:500; CB1-Rb-Af380, Frontier Institute, Hokkaido, Japan) in 4% goat serum. Sections were washed 3 × 5 min in 0.2% PBS-TX and incubated for 1 h at room temperature in dark with secondary antibody (goat anti-rabbit Alexa Fluor 488, 1:1000; Invitrogen, Germany). After a 5-min wash in 0.2% PBS-TX, sections were counterstained with 4′,6-diamidino-2-phenylindole (DAPI) and rinsed in PBS. Sections were then mounted on SuperFrostPlus slides (Menzel, Braunschweig, Germany) and coverslipped with Mowiol 4-88 (Calbiochem, Germany). Fluorescence labeling was visualized and photographed using a confocal laser-scanning microscope (Zeiss Axiovert LSM 710; Carl Zeiss, Oberkochen, Germany) with a 10× or 40× objective. Maximum intensity projections were made of 20-µm *z*-stacks containing six images. Identical exposure settings were used for images that show the same region in brains of different genotypes and brightness and contrast were adjusted identically for these sets of images.

### Western blot

Adult male mice (3.9–4.4 months) were decapitated under isoflurane anesthesia, and brains were isolated and stored at −80 °C. Brains were thawed and hippocampi isolated in ice-cold 50 mM Tris–HCl pH 7.4 and stored at −80 °C. Lysates were prepared using RIPA buffer (50 mM Tris–HCl pH 7.4, 150 mM NaCl, 0.1% SDS, 0.1% sodium deoxycholate, 1% NP-40) containing protease inhibitors (Complete, Roche Applied Science, Germany). Lysis was performed for 60 min at 4 °C, followed by sonication with five pulses at 50% force with a Sonopuls HD60 sonicator (Bandelin, Berlin, Germany). After centrifugation to sediment cellular debris, 20 µg of protein (as measured by Bradford assay) were mixed with 1.5× Laemmli reducing sample buffer. Samples were denatured for 5 min at 60 °C, separated by 10% SDS–PAGE, and then transferred onto nitrocellulose membranes (Protran, Whatman; GE Healthcare, Germany). After blocking in 5% non-fat dry milk, the membrane was incubated overnight at 4 °C; first with rabbit anti-CB1 primary antibody (1:1000; ImmunoGenes AG, Budakeszi, Hungary) and then reprobed with rabbit anti-actin as loading control (1:2000; 04-1040, Millipore, Germany). Antibodies were detected by goat anti-rabbit IgG horseradish peroxidase-conjugated secondary antibody (1:5000; Dianova, Hamburg, Germany) followed by ECL Prime western blotting detection reagent (GE Healthcare). Chemiluminescence was visualized with the Peqlab FUSION-SL Advance 4.2 MP analyzer (Vilber Lourmat, Eberhardzell, Germany) and quantified by densitometric analysis using Bio1D 15.02 software (Vilber Lourmat). Densitrometric data were related to actin and normalized to CB1-RS values.

### Electrophysiological recordings

Adult male mice (3–6 months) were anaesthetized with isoflurane (2.5% in O_2_) and decapitated. Parasagittal hippocampal or coronal amygdala slices (300 µm thick) were prepared as described previously (Lange et al. 2014). Patch electrodes were made of borosilicate glass (GC150T-10; Harvard Apparatus Ltd, Edenbridge, UK). The pipette-resistance was between 2.0 and 2.7 MΩ. The intracellular solution contained (in mM): K-gluconate 100, KCl 50, CsCl 10, HEPES 10, EGTA 0.2, MgCl_2_ 1, MgATP 1, and Na_2_GTP 0.3. The pH was adjusted to 7.30 with KOH. Artificial cerebrospinal fluid (ACSF) was used as extracellular solution and contained (in mM): NaCl 120, KCl 2.5, NaH_2_PO_4_ 1.25, MgSO_4_ 2, CaCl_2_ 2, and glucose 20. The pH was adjusted to 7.30 by gassing with carbogen (95% O_2_, 5% CO_2_). The series resistance *R*_S_ was between 6 and 14 MΩ, was monitored throughout the experiments, and recordings with higher or fluctuating *R*_S_ were discarded. Electrophysiological data were measured with an EPC10-double amplifier (HEKA, Germany) at a sampling rate of 10 kHz and analyzed offline with Clampfit10 software (Molecular Devices Corporation, Sunnyvale, CA, USA). Recordings of depolarization-induced suppression of inhibition (DSI) and excitation (DSE) were performed as described previously (Ruehle et al. 2013). Whole-cell recordings were obtained under voltage-clamp conditions from single neurons at a holding potential of −60 mV. Postsynaptic currents (PSC) were evoked from glutamatergic excitatory (eEPSC) and GABAergic inhibitory (eIPSC) synapses by a bipolar tungsten stimulation electrode. For the hippocampal CA1 region, the stimulation electrode was placed in the stratum radiatum for stimulation of the Schaffer collaterals. For the basolateral amygdala (BLA) and central amygdala (CeA), the stimulation electrode was placed within the local neuropil of the BLA or CeA (~100 μm from the recorded neuron), respectively. Latencies relative to the stimulation artefact were calculated. Latencies were between 0.8 and 4.0 ms, and recordings with higher latencies were discarded. For all DSE and DSI measurements, the stimulation strength was set to evoke ~50% of the maximal electrically induced PSC amplitude. DSE and DSI tests consisted of 30 stimuli (at 0.2 Hz) before and 60 stimuli after postsynaptic depolarization from −60 to 0 mV. The duration of depolarization was 10 s. Evoked responses were normalized to the mean at baseline, and the intensity of suppression was calculated using the mean of five electrically evoked responses before the depolarization and five evoked responses recorded immediately after depolarization.

### Behavioral testing

Adult male mice [2–6 months (average 3.3) at the start of behavioral tests] were individually housed two weeks before behavioral testing to avoid confounding influences of social status, and animals were handled briefly on 2–3 occasions prior to the first test. Animals that were single-housed from weaning on were 2 of 66 CB1-RS, 8 of 71 GABA-CB1-RS and 2 of 65 Stop-CB1 mice. The inclusion of these single-housed mice did not alter the conclusions of the statistical analyses. Independent batches of animals (each containing CB1-RS, Stop-CB1 and GABA-CB1-RS mice) were subjected to a number of in vivo tests over the course of several weeks. The order in which these were performed was determined by the test aversiveness, finishing with the most aversive tests. The order of the tests was as follows: elevated plus-maze, open field and light/dark tests on separate days in 1 week; either cued fear conditioning with extinction or pain-threshold; induction of excitotoxic seizures. All behavioral tests were conducted during the light phase by the same experimenter who was blind to mouse genotype throughout the entire course of the analysis.

### Elevated plus-maze

The elevated plus-maze (EPM) test was performed as described previously (Ruehle et al. 2013) on a maze with arms of 35 cm long and 6 cm wide, elevated 100 cm above the floor. Open arms were surrounded by 3-mm high ledges, while closed arms were surrounded by 20-cm high walls. Light intensity in the open arms was set at 90–140 lx. Mice were placed in the center of the maze, facing a closed arm, and allowed to freely explore the maze for 5 min. After each trial, the plus-maze was cleaned with 70% ethanol. Time spent in the arms was tracked by EthoVision software (Noldus, Wageningen, The Netherlands) and entries of all four paws into each arm were scored manually by a trained observer blind to the genotype. The percentage of time and entries in the open arm were calculated relative to those in all arms.

### Open field

Mice were placed in the center of an illuminated [90–100 lx white box (40 × 40 cm)] and allowed to freely explore the open field for 10 min. After each trial, the open field was cleaned with 70% ethanol. Distance traveled by the animals was tracked by EthoVision software.

### Light/dark test

The light/dark (LD) test was performed as described previously (Ruehle et al. 2013) in a box with a 13 × 39 cm lidded dark compartment and a 26 × 39 cm light compartment connected by a 5 × 5 cm entrance. Light intensity in the middle of the light compartment was set at 90–100 lx. Mice were placed in the dark compartment and then allowed to freely explore the light/dark box for 5 min. After each trial, the light/dark box was cleaned with 70% ethanol. A trained observer blind to the genotype manually scored the latency to first enter the light compartment with all four paws, time spent in the light, and number of entries in the light.

### Cued fear conditioning and extinction

Cued fear conditioning with extinction was performed as described previously (Ruehle et al. 2013). Animals were conditioned in context A (Med Associates conditioning chamber; 15 × 20 cm rectangular with grid floor, cleaned with 1% acetic acid). The session started when the house light (25 lx) was switched on. After 3 min, a 20 s tone (80 dB, 9 kHz sine wave, 10 ms rising and falling time) was presented as the conditioned stimulus (CS). The tone coterminated with a single 2-s scrambled electric foot shock of 0.6 mA (2.5–3 times the average pain threshold) as the unconditioned stimulus (US). Mice were returned to their home cages 60 s after the end of the CS–US presentation. A mild conditioning procedure was chosen to optimize detection of a CB1-related phenotype in accordance with previous studies. This included a single tone-shock pairing to avoid confounding by genotype differences in habituation to repeated pain stimuli (Azad et al. 2005; Ruehle et al. 2012) and a relatively low shock intensity to induce intermediate freezing levels (Marsicano et al. 2002; Kamprath et al. 2006, 2011; Dubreucq et al. 2012; Ruehle et al. 2013), allowing detection of deviations in freezing responses in both directions. On days 1, 2, 3, and 10 after the conditioning day, conditioned mice were exposed to an extinction session in context B (custom-made Plexiglas cylinders; 15 cm diameter, with fresh bedding, cleaned with 70% ethanol). The session started when the house light (5 lx) was switched on. After 3 min, a 200-s continuous tone (CS, same settings as in conditioning) was presented. Mice were returned to their home cages 60 s after the end of the CS presentation. Animals were tracked using EthoVision software. Immobility was scored with the EthoVision immobility filter set at a threshold optimized for the type of cameras used (i.e., 0.6 or 0.8% change of the pixels representing the mouse), with averaging over two consecutive frames (25 frames/s). Immobility before, during, and after the tone were measured, and the latter two were adjusted for the former. This adjustment for baseline immobility (before the tone) was done by reducing all immobility responses by the average immobility before tone onset. This subtraction method was shown to be appropriate for “weak” tone conditioning [Jacobs et al. 2010; “weak” was defined as 1 tone-shock pairing (20 s, 2800 Hz, 85 dB) and (2 s, 0.5 mA), similar to our conditions]. Within-session extinction was defined as the reduction in immobility over the 200-s CS presentation (Riebe et al. 2012), relative to the individual initial response. Between-session extinction could be assessed by comparing the initial responses between days. As an additional control for initial fear learning, a batch of animals was included with both conditioned and non-conditioned mice (only CS exposure during conditioning, no US foot shock) for each genotype. The initial conditioned response was expressed as the difference of immobility during the first 20 s of the CS tone on day 1 (adjusted for baseline immobility) between conditioned and unconditioned animals.

### Pain threshold

A separate batch of mice, not exposed to cued fear conditioning, was tested for pain threshold. Animals were placed into the conditioning chamber and, after 2-min habituation with the houselight on, 1-s scrambled electric foot shocks of rising intensity (in 0.05-mA steps) were applied every 30 s. Pain threshold was scored as the first shock intensity that the mice responded to by either jumping or vocalization.

### Induction of acute excitotoxic seizures

Acute epileptiform seizures were induced by intraperitoneal injection of kainic acid (20 mg/kg, Sigma–Aldrich, Germany) dissolved in 0.9% saline in a volume of 10 ml/kg body weight as previously described (Ruehle et al. 2013). Before kainic acid injection, the animals were given a light isoflurane inhalation anesthesia to reduce injection stress. A trained observer blind to the genotype of the mice monitored the severity of seizures for 2 h and scored every 15 min according to the modified Racine scale used before (Schauwecker and Steward 1997; Marsicano et al. 2003).

### Statistical analysis

Relative binding levels of receptor autoradiography were analyzed using repeated measures ANOVA for genotype with brain region as within-subject factor, followed by simple effects analysis per region when a significant interaction between genotype and brain region was found or by Tukey post hoc test when no interaction between genotype and brain region was found. Relative protein levels from western blot were analyzed using one-way ANOVA for genotype. Electrophysiological measurements of DSE and DSI were analyzed per genotype using a paired *t* test to compare between time-points (before vs after depolarization). For behavioral parameters, after an initial analysis to exclude major differences between independently tested batches of all groups in an experiment, data of the different batches were pooled either directly or after normalization to the control group (CB1-RS) for each batch. Anxiety measures from the EPM and distance travelled in the open field were analyzed using one-way ANOVA for genotype, with Welch correction where Levene’s test indicated inhomogeneity for variances. Univariate ANCOVA for genotype was used to analyze anxiety measures from the LD test, with distance from the open field as a covariate. Parameters of fear conditioning and kainic acid-induced seizure scores were analyzed using repeated measures ANOVA for genotype with time as within-subject factor. The Greenhouse–Geisser correction was used if the assumption of sphericity was not met. Survival following kainic acid injections was evaluated by the Kaplan–Meier method and analyzed by the log-rank test, followed by pairwise Mantel–Cox log-rank tests. Spontaneous deaths throughout the experiments were analyzed using Fisher’s exact test. Significant genotype effects from ANOVA were further analyzed using Tukey or Games–Howell post hoc analysis for multiple comparisons as appropriate. After a significant interaction of time with genotype in repeated measures ANOVA, simple effects for genotype were determined for each time-point/interval using Sidak correction. Statistical analyses were conducted using SPSS Statistics Software for Windows (version 22; IBM, Chicago, IL, USA). Data are expressed as mean ± SEM, and statistical significance was defined as *P* < 0.05. Number of animals (and cells) used for all measures are provided in figure legends or on graphs.

## Results

### Excision of the stop-cassette in Dlx-positive neurons restores CB1 receptor function in forebrain GABAergic neurons (GABA-CB1-RS)

To obtain a selective rescue of the endogenous CB1 receptor in forebrain GABAergic neurons (GABA-CB1-RS), Stop-CB1 mice with a floxed stop-cassette in the 5′ UTR of the CB1 coding sequence (Ruehle et al. 2013) were crossed with a mouse line expressing Cre recombinase under the control of the regulatory elements of the *Dlx5*/*Dlx6* enhancer (Monory et al. 2006). Stop-CB1 mice, lacking functional CB1 receptors, were used as a knockout-like control and CB1-RS mice, with a global rescue of the CB1 receptor in all cell types, were used as a wildtype-like control. Both control groups were included in all analyses to monitor CB1 receptor functionality. Sufficiency of the rescue for restoration of the phenotype was defined as described previously (Ruehle et al. 2013). If the genotype of interest (GABA-CB1-RS) showed a significant difference from Stop-CB1 and no significance compared with the CB1-RS control group, the rescued population of CB1 receptor was considered to be “fully sufficient” for the function under investigation. Values intermediate between Stop-CB1 and CB1-RS (with significance from either both or none), were considered “partly sufficient”. No difference from Stop-CB1 together with a significant difference from CB1-RS controls was interpreted as “no sufficiency”.

**Fig. 1:**
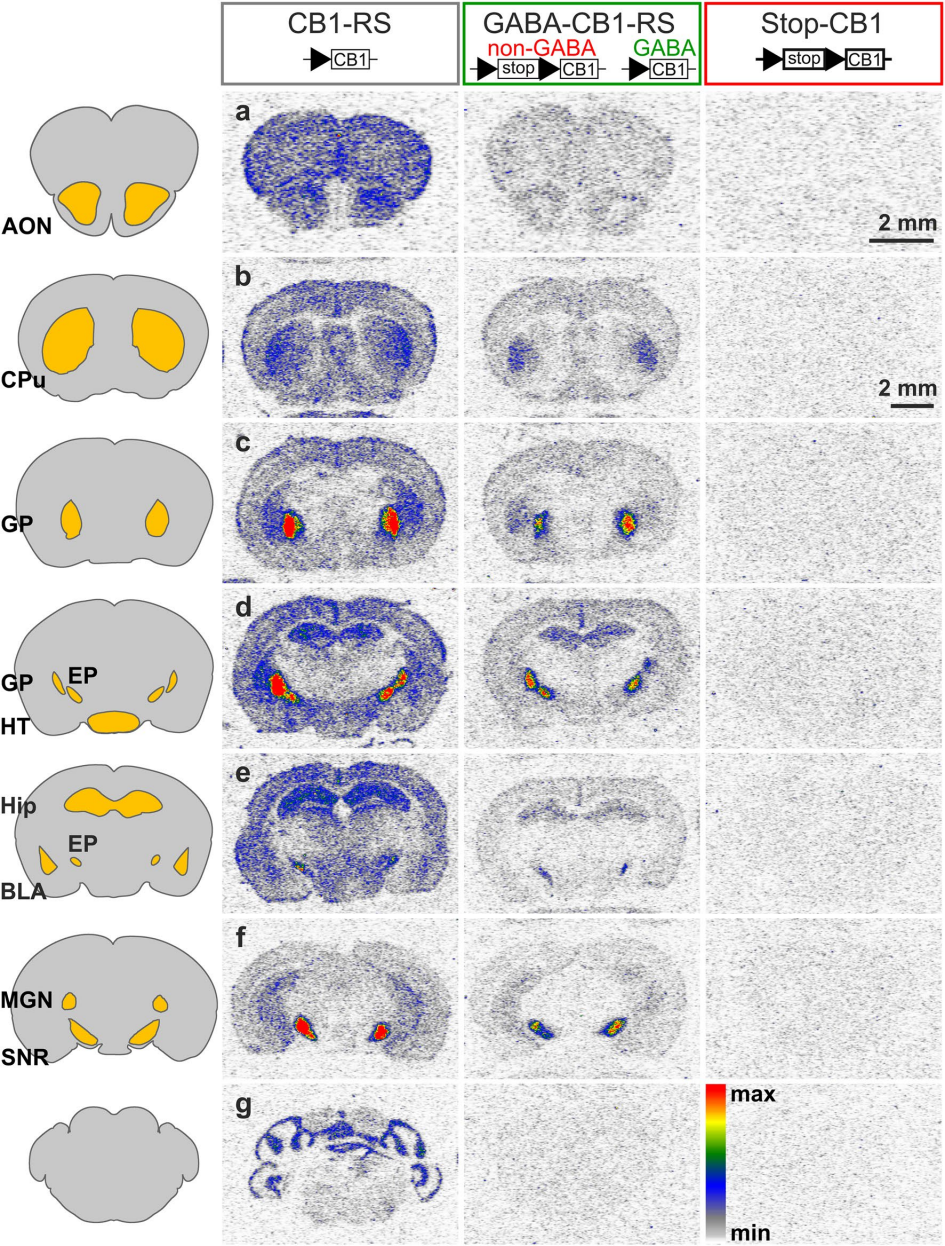
Rescue of the CB1 receptor in forebrain GABAergic neurons restores a substantial amount of functional CB1 receptor protein in various brain regions. Autoradiograms of CB1 receptor ligand binding using the CB1 receptor agonist ^3^H-CP 55,940 on coronal sections of CB1-RS, GABA-CB1-RS, and Stop-CB1 brains, with schematic diagrams of the mouse brain depicting the approximate location of brain regions where the signal was quantified, according to the mouse brain atlas (Franklin and Paxinos 2007). Distance from bregma (in mm): **a** 2.6; **b** 0.3; **c** −0.5; **d** −1.3; **e** −1.6; **f** −3.2; **g** −5.2. *Black triangles*, loxP sites; *white box* (stop), stop cassette; *white box* (CB1), CB1 receptor open reading frame. *AON* anterior olfactory nucleus, *BLA* basolateral amygdala, *CPu* caudate putamen, *EP* entopeduncular nucleus, *GP* globus pallidus, *Hip* hippocampus, *HT* hypothalamus, *MGN* medial geniculate thalamic nucleus, *SNR* substantia nigra. *Scale bars* 2 mm (in **a** for **a**, in **b** for **b**–**g**)

**Fig. 2:**
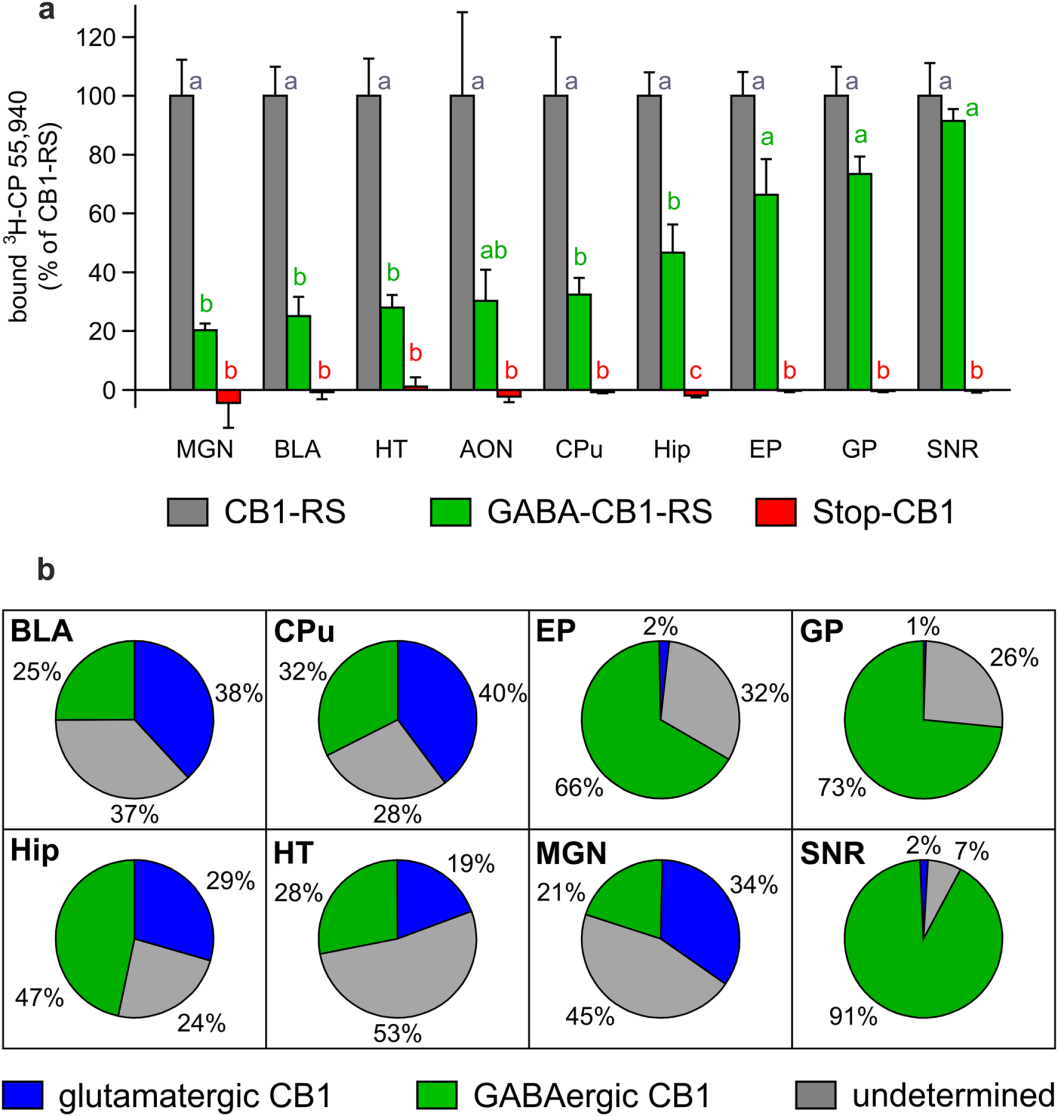
Relative contribution of two neuronal subpopulations to the total amount of functional CB1 receptor protein in various brain regions. **a** Quantification of signal intensity per brain region expressed relative to that in CB1-RS mice (*n* = 3 brains per group); values of *columns* labeled with the *same letter* (*a*–*c*) are not significantly different from each other in repeated measures ANOVA followed by simple effects analysis per brain region with Sidak correction; data are expressed as mean + SEM; details of statistical analysis in supplementary Table S2. **b** Agonist binding levels relative to those in CB1-RS controls, compared between Glu-CB1-RS (as determined in Ruehle et al. 2013 and supplementary Fig. S1) and GABA-CB1-RS (as determined in Fig. 2a) in eight brain regions. *AON* anterior olfactory nucleus (mean of 6 sections/animal in **a**), *BLA* basolateral amygdala (6), *CPu* caudate putamen (9), *EP* entopeduncular nucleus (7), *GP* globus pallidus (4), *Hip* hippocampus (10), *HT* hypothalamus (9), *MGN* medial geniculate thalamic nucleus (7), *SNR* substantia nigra (6). *Blue* agonist binding on dorsal telencephalic glutamatergic neurons; *green* agonist binding on forebrain GABAergic neurons; *grey* (undetermined) agonist binding not present on either dorsal telencephalic glutamatergic neurons or forebrain GABAergic neurons

The distribution of CB1 receptors was visualized by the binding of the radioactively-labeled synthetic CB1 receptor agonist ^3^H-CP 55,940 in coronal brain sections of GABA-CB1-RS mice and their Stop-CB1 and CB1-RS controls. Compared with CB1-RS mice, cannabinoid binding was partially restored in GABA-CB1-RS mice in many areas throughout the brain, with no distinguishable signal in Stop-CB1 mice (Fig. 1). Not surprising for a forebrain-specific rescue, the hindbrain and cerebellum were practically void of any signal in GABA-CB1-RS brains. For several brain regions, the amount of binding was quantified relative to that in the according CB1-RS region (Fig. 2a). Despite the small sample size, an overall analysis (repeated measures ANOVA) showed a significant interaction between genotype and region (*P* < 0.001, all detailed statistics in supplementary Table S2), and subsequent simple effect analysis revealed distinct degrees of rescue in the different regions. In the thalamus and amygdala, the rescue seen in GABA-CB1-RS brains was relatively modest, with intermediate levels in the hippocampus, whereas the strong signal in the basal ganglia was largely GABAergic in origin (Fig. 2a). By combining the present data with those previously reported for the dorsal telencephalic glutamatergic CB1 receptor rescue mouse, Glu-CB1-RS (Ruehle et al. 2013), the relative contribution of these two neuronal populations to the total amount of CB1 receptors can be assessed for distinct brain regions. The glutamatergic contribution prevails in amygdalar, striatal, and thalamic regions, with a larger GABAergic contribution in the hypothalamus, hippocampus, and basal ganglia (Fig. 2b). In all tested regions except the substantia nigra, cell populations other than the dorsal telencephalic glutamatergic neurons and the forebrain GABAergic neurons provide substantial amounts of CB1 receptor.

As they are centrally involved in the endocannabinoid-mediated regulation of emotional behaviors and seizure susceptibility (Soltesz et al. 2015; Tovote et al. 2015; Lutz et al. 2015), hippocampus and amygdala were further investigated after genetic rescue. CB1 protein distribution and the specific functional rescue of the archetypical CB1-mediated retrograde suppression, DSI and DSE, were verified in GABA-CB1-RS mice and their controls.

**Fig. 3:**
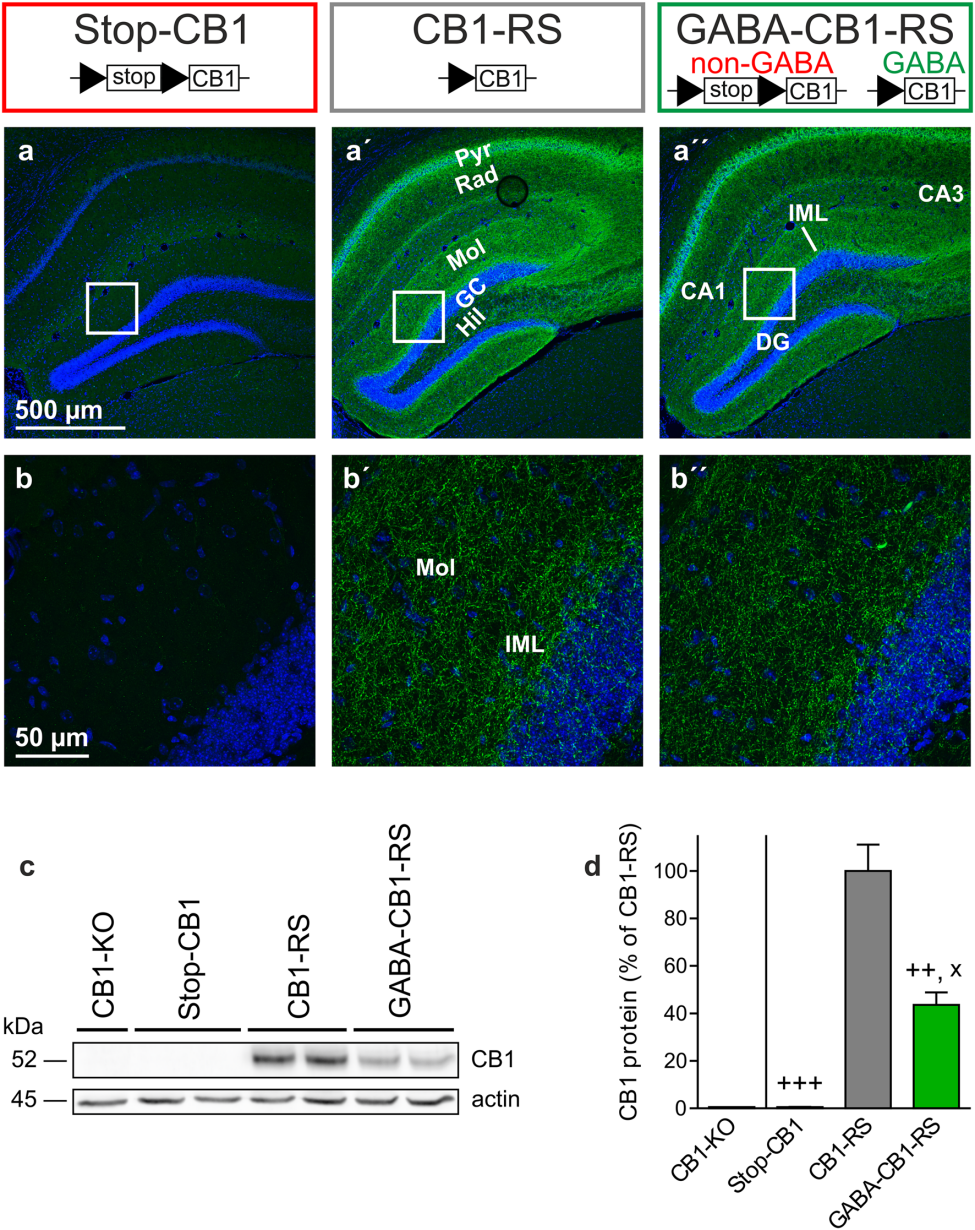
Rescue of the CB1 receptor in forebrain GABAergic neurons: selective restoration of hippocampal protein. **a** CB1 receptor immunostaining (*green*) and nuclear staining with DAPI (*blue*) in the hippocampus of Stop-CB1, CB1-RS and GABA-CB1-RS mice (representative of 2–3 animals per genotype). **b** Higher magnification micrographs as indicated in **a**, showing very similar fiber-like staining (also in the inner third of the molecular layer) of CB1-RS and GABA-CB1-RS mice. No specific staining was detected in Stop-CB1 mice, similar to CB1-RS sections that were processed without primary anti-CB1 antibody (not shown). **c** Representative western blot of hippocampal homogenates, stained with antibodies against the CB1 receptor and actin. No CB1 receptor signal was detected in a CB1-KO mouse and in Stop-CB1 mice, with lower intensity of labeling in GABA-CB1-RS mice than in CB1-RS control mice. **d** Quantification of CB1 receptor protein normalized to actin protein, shown relative to that in CB1-RS mice (*n* = 3 animals per genotype); one CB1-KO sample is shown as a reference. Data are expressed as mean ± SEM. *CA1/3* cornu ammunis region 1/3, *DG* dentate gyrus, *GC* granule cell layer, *Hil* hilar region, *IML* inner molecular layer, *Mol* stratum moleculare, *Pyr* CA1/CA3 pyramidal cell layer, *Rad* stratum radiatum. Significant differences in protein levels (**d**) were determined with one-way ANOVA comparing the three groups followed by Tukey multiple comparison test; ^x^ *P* < 0.05 vs Stop-CB1; ^++^ *P* < 0.01 vs CB1-RS; ^+++^ *P* < 0.001 vs CB1-RS; details of statistical analysis in supplementary Table S2

**Fig. 4:**
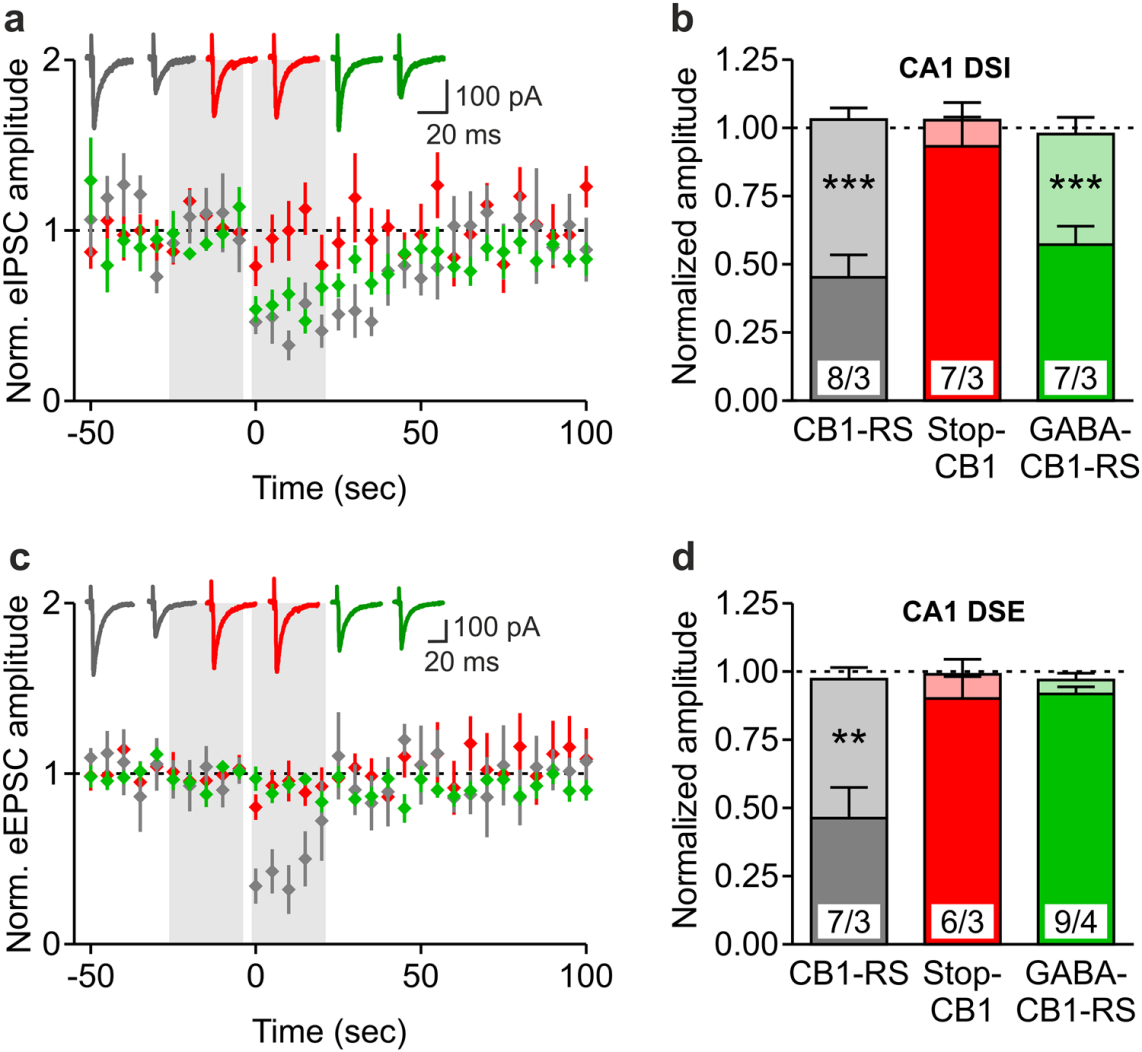
Rescue of the CB1 receptor in forebrain GABAergic neurons: selective restoration of hippocampal synaptic function. **a, c** CB1-mediated depolarization-induced suppression of inhibition (DSI) (**a**) and excitation (DSE) (**c**) in hippocampal CA1 pyramidal neurons of CB1-RS (*grey*), Stop-CB1 (*red*) and GABA-CB1-RS (*green*) mice. Averaged normalized eIPSC and eEPSC amplitudes, before and after postsynaptic depolarization (−60 to 0 mV; 10 s duration; at time-point zero). Original traces illustrate eIPSCs or eEPSCs immediately before and after the postsynaptic depolarization. **b, d** Summary bar graphs of the five last evoked responses before (*light colors*) and the five first evoked responses after depolarization (*darker colors*), showing the magnitude of depression. Excitatory and inhibitory postsynaptic currents are both significantly depressed at the post-depolarization time-point in CB1-RS mice, but not in Stop-CB1 mice. GABA-CB1-RS mice show significant DSI, but not DSE. Data are expressed as mean ± SEM; numbers of cells/animals are indicated in **b** and **d**. DSI (**b**) and DSE (**d**) were analyzed using paired *t* test comparing the five last evoked responses before and the five first evoked responses after depolarization; **P < 0.01; ***P < 0.001; details of statistical analysis in supplementary Table S2

In the hippocampus of GABA-CB1-RS mice, immunohistochemistry revealed mesh-like patterns of CB1 receptor protein that were very similar to those in control CB1-RS mice, whereas no staining could be detected in the brains of Stop-CB1 mice (Fig. 3a). This expression pattern is consistent with previous reports, where GABAergic neurons were shown to contain a large majority of brain CB1 receptor protein (Monory et al. 2006; Bellocchio et al. 2010; Gutiérrez-Rodríguez et al. 2017). Also, we previously showed only weak staining in the conditional rescue in dorsal telencephalic glutamatergic neurons (Ruehle et al. 2013). Even the expression in the inner third of the molecular layer of the dentate gyrus, where the glutamatergic contribution to CB1 receptor protein is highest (Monory et al. 2006; Ruehle et al. 2013), could not be readily distinguished by this method (Fig. 3b). These results fit to the GABAergic-only staining reported in cortex in GABA-CB1-RS mice (de Salas-Quiroga et al. 2015). To obtain a quantitative measurement of hippocampal CB1 receptor protein expression, western blot analysis was performed. Fitting with the binding data, western immunoblotting for the receptor in hippocampal homogenates of these animals showed a clearly reduced expression of CB1 receptor protein in GABA-CB1-RS mice compared with that in CB1-RS and again confirmed the absence of CB1 receptor protein in Stop-CB1 mice and in a CB1-KO control (Fig. 3c). A quantitative analysis of the western blot data showed that the amount of CB1 receptor protein in the hippocampus of GABA-CB1-RS mice is around 45% of that in control (CB1-RS) hippocampus (Fig. 3d). To verify both the functionality and specificity of the rescued receptors, we measured CB1 receptor-mediated depolarization-induced retrograde suppression of both glutamatergic (DSE) and GABAergic (DSI) transmission (Wilson and Nicoll 2001; Ohno-Shosaku et al. 2002) in the CA1 of GABA-CB1-RS mice and their wildtype-like and knockout-like controls for CB1 function. As expected, depolarization of the recorded neuron reduced both GABAergic and glutamatergic postsynaptic currents in the CB1-RS wildtype-like controls, whereas DSI and DSE were both absent in the Stop-CB1 knockout-like controls (Fig. 4). In GABA-CB1-RS slices, the functionality of the rescued CB1 receptors was demonstrated by the restoration of DSI (Fig. 4a, b). Selectivity of the rescue for GABAergic signaling was confirmed by the concomitant absence of DSE in GABA-CB1-RS slices (Fig. 4c, d).

**Fig. 5:**
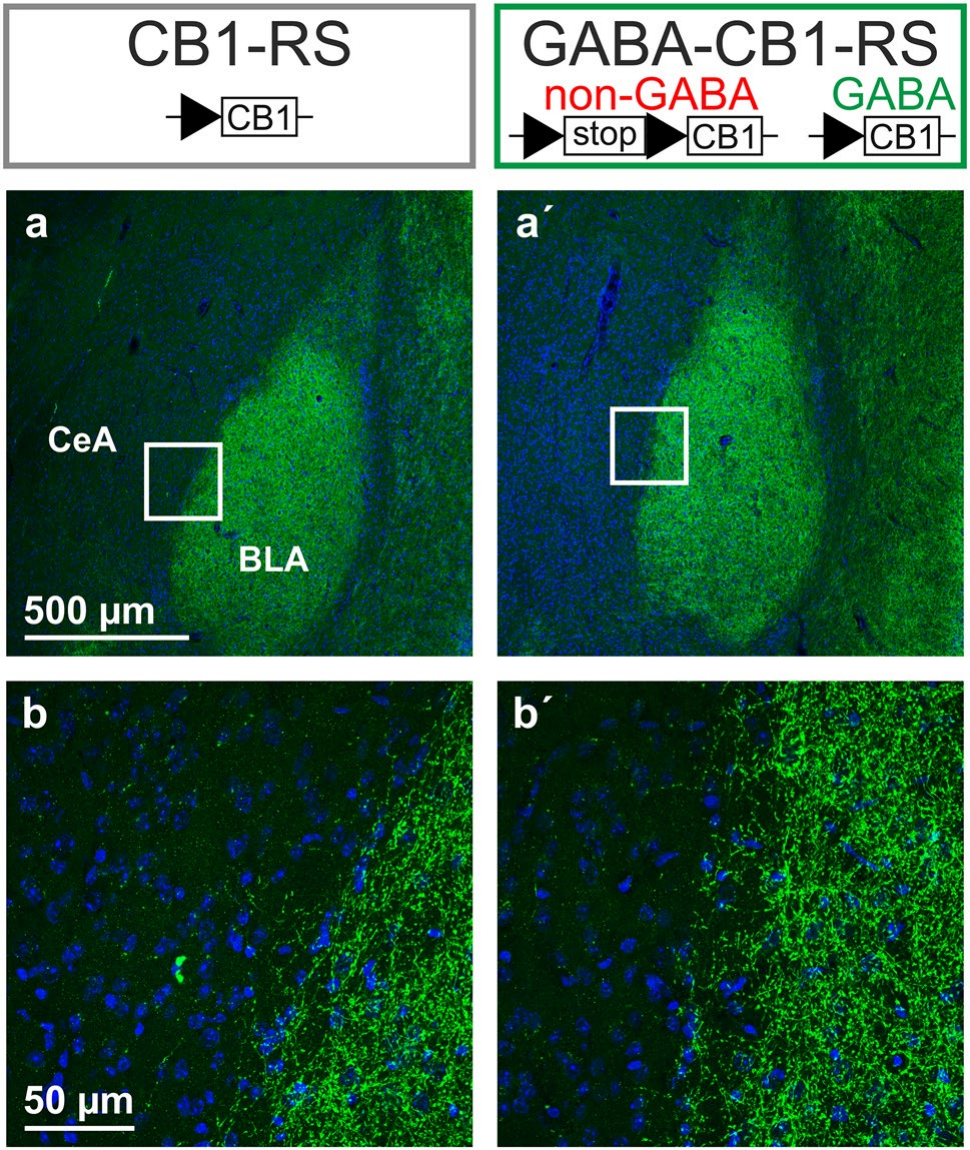
Rescue of the CB1 receptor in forebrain GABAergic neurons: selective restoration of amygdalar protein. **a** CB1 receptor immunostaining (*green*) and nuclear staining with DAPI (*blue*) in the amygdala of CB1-RS and GABA-CB1-RS mice (representative of 2–3 animals per genotype). No specific staining was detected in Stop-CB1 mice (not shown), similar to CB1-RS sections that were processed without primary anti-CB1 antibody (not shown). **b** Higher magnification micrographs as indicated in **a**, showing dense mesh-like patterns of CB1 immunoreactivity in the basolateral amygdala (BLA) of both genotypes, with sparser fibers in the central amygdala (CeA)

**Fig. 6:**
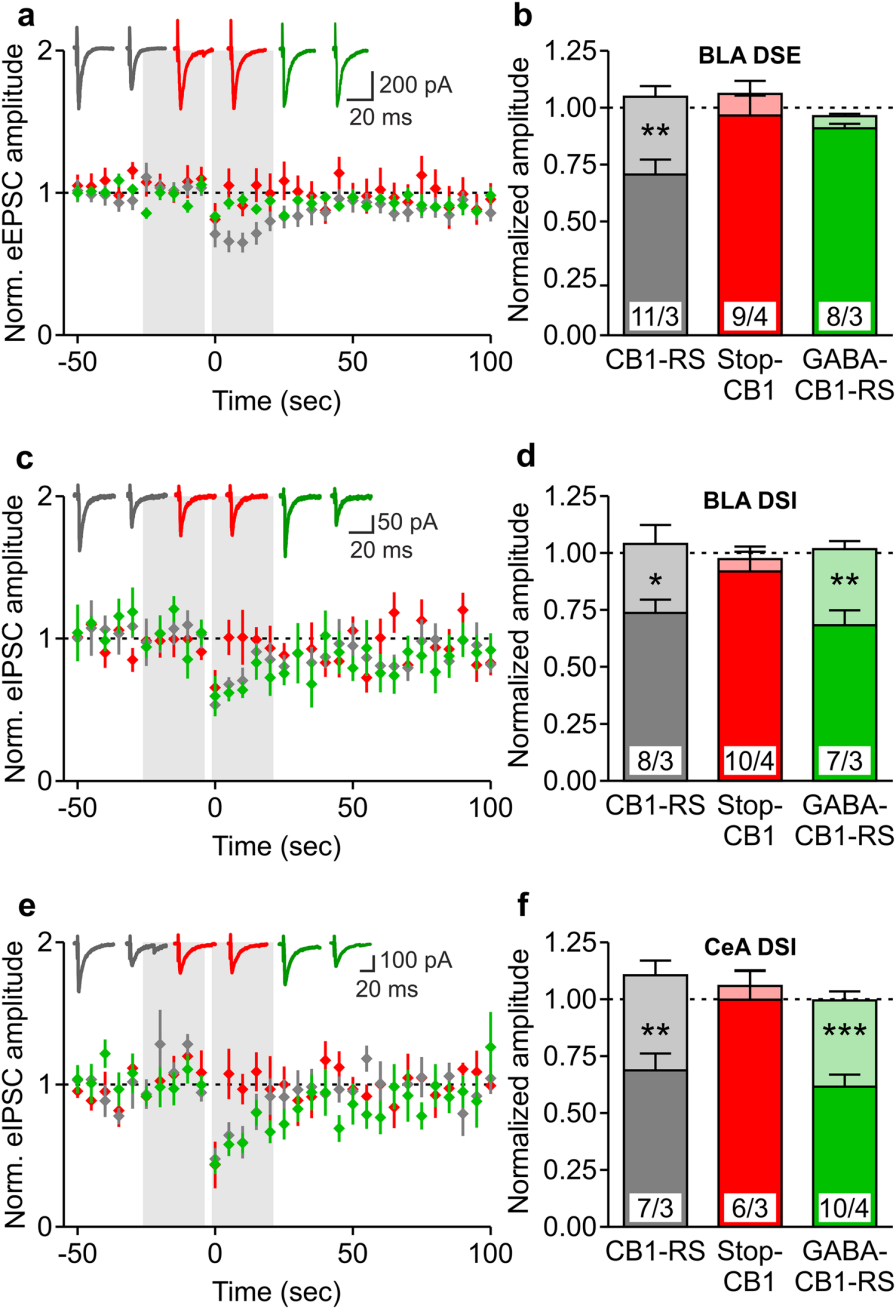
Rescue of the CB1 receptor in forebrain GABAergic neurons: selective restoration of amygdalar synaptic function. **a, c, e** CB1-mediated depolarization-induced suppression of excitation (DSE) (**a**) and inhibition (DSI) in basolateral amygdala (BLA) (**c**) and DSI in central amygdala (CeA) (**e**) principal neurons of CB1-RS (*grey*), Stop-CB1 (*red*) and GABA-CB1-RS (*green*) mice. Averaged normalized eEPSC and eIPSC amplitudes, before and after postsynaptic depolarization (−60 to 0 mV; 10 s duration; at time-point zero). Original traces illustrate eEPSCs or eIPSCs immediately before and after the postsynaptic depolarization. **b, d, f** Summary bar graphs of the five last evoked responses before (*light colors*) and the five first evoked responses after depolarization (*darker colors*), showing the magnitude of DSE (**b**) and DSI (**d, f**). Excitatory and inhibitory postsynaptic currents are both significantly depressed at the post-depolarization time-point in CB1-RS mice, but not in Stop-CB1 mice. GABA-CB1-RS mice show significant DSI, but not DSE. Data are expressed as mean ± SEM; numbers of cells/animals are indicated in **b, d, f**. *P < 0.05; **P < 0.01; ***P < 0.001 in paired *t* test comparing the five last evoked responses before and the five first evoked responses after depolarization; details of statistical analysis in supplementary Table S2

In the amygdala, the expression patterns of CB1 receptor protein detected by immunohistochemistry were also very similar between GABA-CB1-RS mice and the global rescue CB1-RS (Fig. 5a). Again, no signal was detected in Stop-CB1 mice (data not shown). At a higher magnification, apart from the strong staining in the BLA, sparse fibers were detected in the CeA in both GABA-CB1-RS and CB1-RS sections (Fig. 5b). To verify both the functionality and specificity of the rescued receptors in the amygdala, DSI and DSE were recorded in brain slices prepared from GABA-CB1-RS mice and their CB1-RS wildtype-like and Stop-CB1 knockout-like controls. DSI and DSE were confirmed to both be present in CB1-RS mice and absent in Stop-CB1 mice (Fig. 6). In the BLA of GABA-CB1-RS, we verified the absence of DSE (Fig. 6a, b) and the presence of DSI (Fig. 6c, d). Additionally, DSI was restored in the CeA of GABA-CB1-RS mice (Fig. 6e, f); a region where CB1 protein is also reported to be present (Patel et al. 2005; Kamprath et al. 2011; Ramikie and Patel 2012; Ruehle et al. 2013; Ramikie et al. 2014; and Fig. 5). After the GABAergic rescue of the CB1 receptor had been verified, GABA-CB1-RS mice and their CB1-RS and Stop-CB1 controls were tested for seizure susceptibility and for their response in two paradigms of aversive emotional behavior.

### GABAergic CB1 receptor is not sufficient for protection against chemically induced seizures

**Fig. 7:**
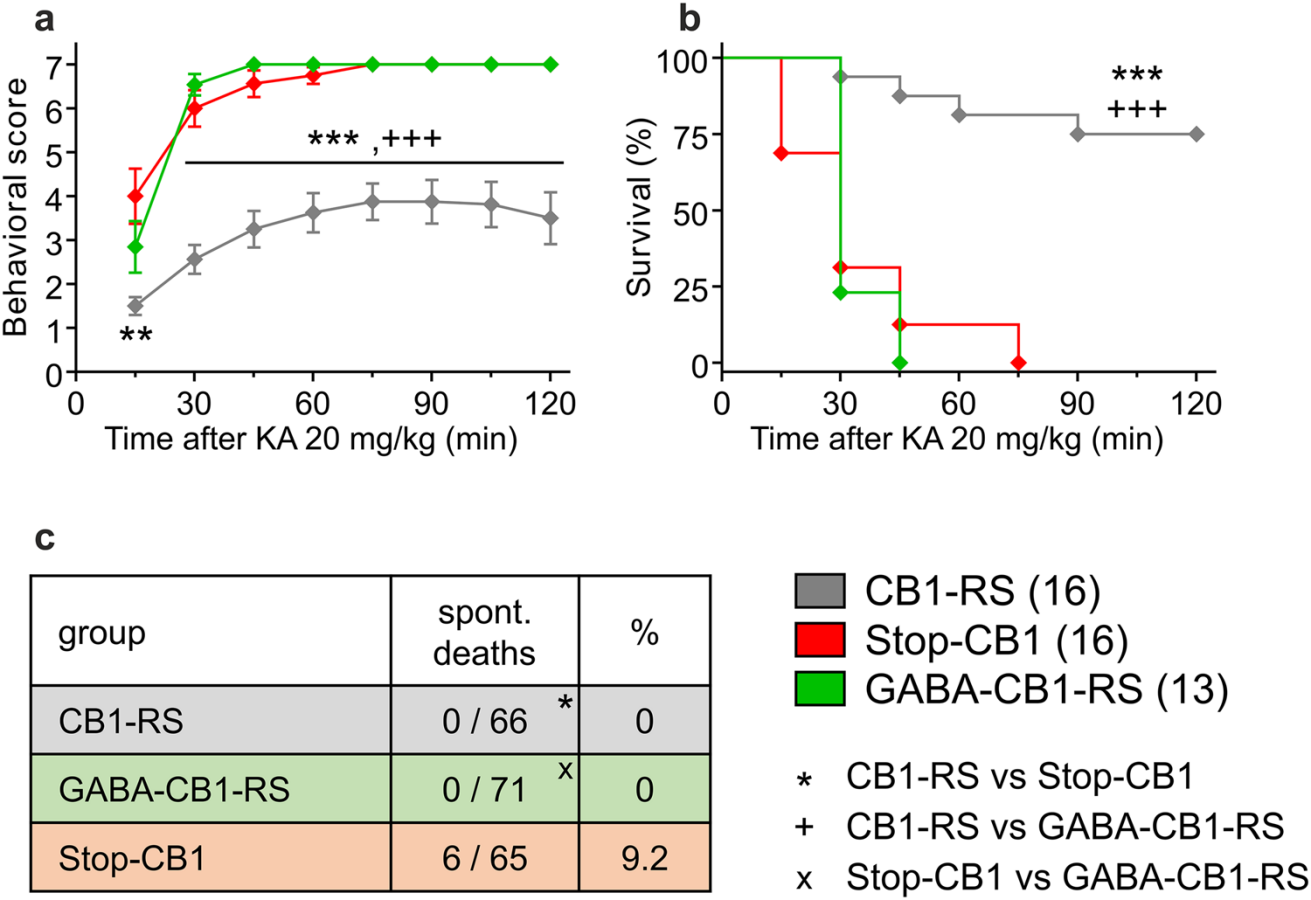
No sufficiency of rescue of the CB1 receptor specifically in forebrain GABAergic neurons for protection against chemically induced seizures. Susceptibility to seizures induced by the excitotoxin kainic acid (KA, 20 mg/kg) was higher in both Stop-CB1 and GABA-CB1-RS mice than in CB1-RS control mice, as shown by higher behavioral scores (**a**) and reduced survival (**b**) over a period of 120 min after KA injection. However, spontaneous deaths (**c**) were not observed in CB1-RS or GABA-CB1-RS mice throughout the experiments, only in Stop-CB1 mice. Data are presented as mean ± SEM (**a**) or as Kaplan–Meier survival curves (**b**); animal numbers are indicated. Significant differences in behavioral score (**a**) were determined in repeated measures ANOVA, followed by post hoc simple effect analysis per time-point with Sidak correction; survival after KA injection (**b**) was analyzed using the log-rank test; spontaneous deaths throughout the experiments (**c**) were analyzed using Fisher’s exact test. Significant differences are indicated by *between CB1-RS and Stop-CB1, ^+^between CB1-RS and GABA-CB1-RS, ^x^between Stop-CB1 and GABA-CB1-RS (1, *P* < 0.05; 2, *P* < 0.01; 3, *P* < 0.001); details of statistical analysis in supplementary Table S2

A major phenotype of CB1 null mutants is an increased susceptibility to epileptiform seizures (Marsicano et al. 2003). Whereas CB1 receptors in dorsal telencephalic glutamatergic neurons were found to be both necessary and sufficient for protection against acute excitotoxic kainic-acid induced seizures (Monory et al. 2006; Ruehle et al. 2013), no necessary role was previously found for those in forebrain GABAergic neurons (Monory et al. 2006). Because of the high susceptibility of Stop-CB1 mice and predicted similarly high susceptibility in GABA-CB1-RS mice, we administered a low dose of kainic acid (20 mg/kg instead of 30 mg/kg) to increase the chances to detect possibly small differences in either direction (reduced or increased seizure-susceptibility) between these groups. Although at the first observation, 15 min after kainic acid injection, seizure severity of GABA-CB1-RS mice was intermediate between the low score of CB1-RS mice and the significantly higher score of Stop-CB1 mice, the seizure severity in GABA-CB1-RS mice was never significantly different from that in Stop-CB1 mice (Fig. 7a). After the first time-point, both groups had significantly higher scores than control CB1-RS mice (Fig. 7a). This feature was also reflected in the survival curve; all GABA-CB1-RS survived the first 15 min of the test, but their overall survival was not different from that of Stop-CB1 mice (*P* > 0.9; Fig. 7b). Possibly, CB1 receptors in GABAergic neurons can delay the onset of seizures, but cannot protect against their eventual severity. A small degree of protection is also supported by the number of spontaneous deaths in the different animal groups throughout the experiments. These occurred only in the Stop-CB1 group (Fig. 7c), and all deceased animals were found in a “stretched-out” position, a posture that is also seen in mice dying after excitotoxic seizures. Therefore, although CB1 receptors in forebrain GABAergic neurons did not provide substantial protection against kainic-acid induced seizures, they may still play a distinct role in other types of seizures.

### Innate anxiety-like behavior is largely restored to normal in GABA-CB1-RS mice

**Fig. 8:**
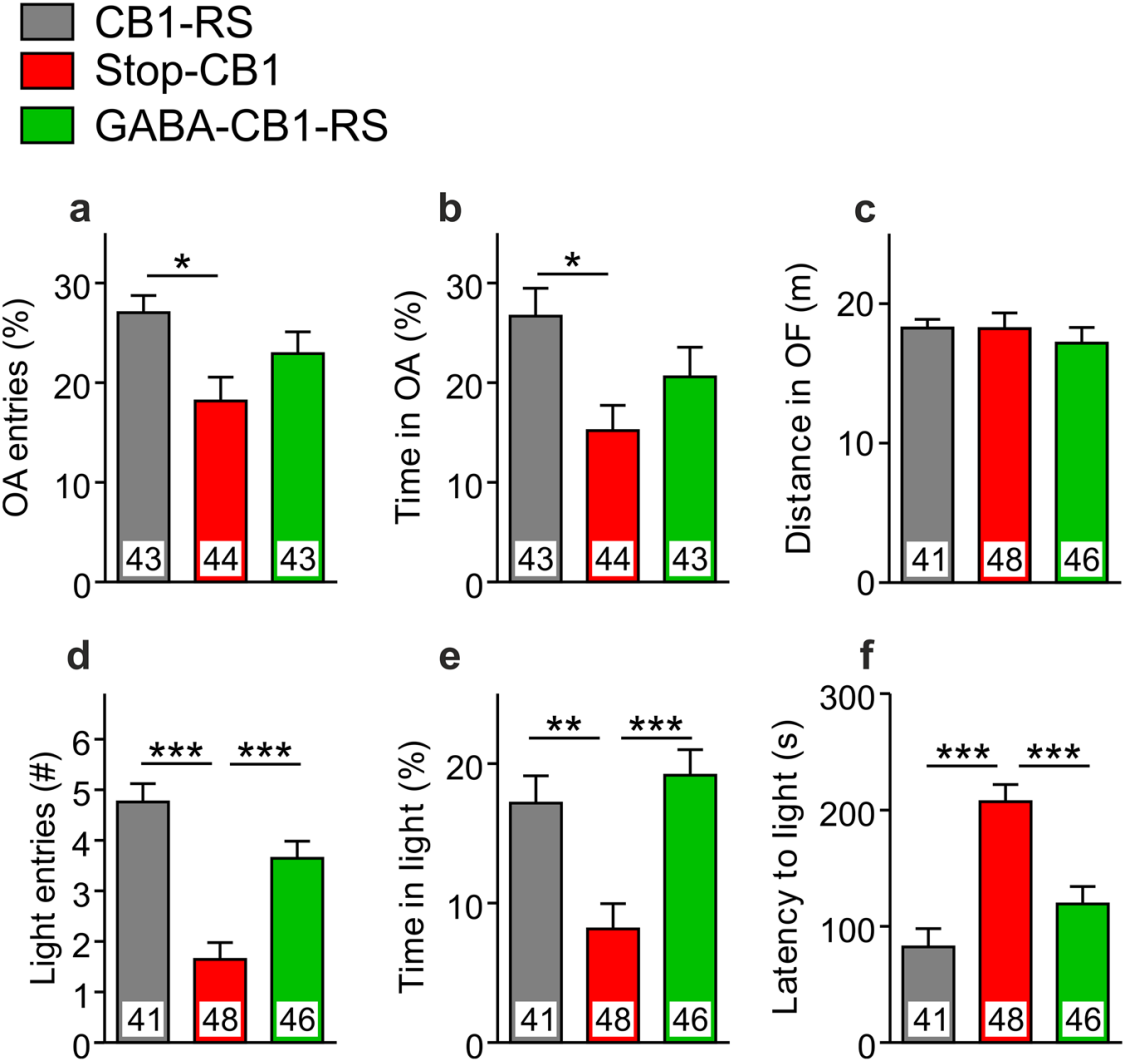
Anxiety-like behavior is largely restored to normal by rescue of the CB1 receptor specifically in forebrain GABAergic neurons. In the elevated plus-maze (EPM) test, **a** the number of entries into the open arms (OA) expressed as percentage of total arm entries and **b** the time spent in the OA (as percentage of time in total arms) were lower in Stop-CB1 animals than in CB1-RS control animals. GABA-CB1-RS mice had intermediate values and did not differ significantly from both other groups, indicating a partial restoration of the anxiogenic phenotype in GABA-CB1-RS mice. **c** Distance traveled in the open field (OF) was similar between the three groups. In the light/dark (LD) test, **d** the number (#) of entries in the light compartment and **e** the percentage of time spent in the light compartment was similar between CB1-RS and GABA-CB1-RS mice and significantly lower in Stop-CB1 mice, pointing to a full restoration of the anxiogenic phenotype in GABA-CB1-RS mice. **f** Latency to the first entry of the light compartment was also similar between CB1-RS and GABA-CB1-RS mice and significantly higher in Stop-CB1 mice, confirming that in GABA-CB1-RS mice the phenotype of CB1 receptor deficiency is completely remedied in this paradigm. Data are expressed as mean + SEM (**a**–**c**) or covariate-adjusted means + SEM (**d**–**f**); animal numbers are indicated in the graphs; *P < 0.05; **P < 0.01; ***P < 0.001 in one-way ANOVA (for EPM and OF) or univariate ANOVA with OF distance as covariate (for LD), followed by Tukey multiple comparison test; details of statistical analysis in supplementary Table S2

Anxiety-like behavior was assessed in the elevated plus-maze (EPM) and the light/dark (LD) test, with open field locomotion as a control for possible confounding by general activity in the latter test. The EPM has internal controls for locomotion, with entries into open arms assessed relative to those in all arms. Locomotion parameters in the EPM did not differ greatly between the groups. Although Stop-CB1 mice covered more distance than GABA-CB1-RS mice (with no significance of either to CB1-RS mice), no group differences occurred in total arm visits or amounts of time in the center and arms of the maze (supplementary Fig. S2). As seen previously in the EPM test under high-light conditions (Haller et al. 2004; Ruehle et al. 2013), CB1 receptor deficient Stop-CB1 mice showed more anxiety-like behavior with fewer entries into the open arms and less time spent in the open arms than CB1-RS mice (Fig. 8a, b). For both parameters, GABA-CB1-RS mice displayed intermediate values, with no significance to either of the two control groups. Distance covered in the open field was similar between the genotypes, excluding confounding influences by general changes in locomotor activity on genotype differences in anxiety-like as well as other behaviors (Fig. 8c). Nonetheless, open-field exploration had a significant effect as a covariate on parameters of the LD test and was, therefore, included in the analysis for that test. In the LD test, Stop-CB1 mice (as expected; Jacob et al. 2009; Ruehle et al. 2013) again showed significantly higher anxiety levels than the CB1-RS wildtype-like controls with fewer entries into the light compartment, less time spent in the light compartment, and a longer latency to first enter the light compartment (Fig. 8d–f). GABA-CB1-RS mice had values very similar to those of CB1-RS mice and significantly different from Stop-CB1 knockout-like controls. Thus, LD-specific anxiety was fully restored in GABA-CB1-RS mice, whereas for EPM-specific anxiety the rescue was partial. Together, these results point to a large degree of sufficiency of the CB1 receptor in forebrain GABAergic neurons to reverse the increase in anxiety-like behavior observed in Stop-CB1 mice.

### GABAergic CB1 receptors convey limited sufficiency for fear extinction

CB1 receptor null mutants exhibit a deficit in the extinction of learned fear (Marsicano et al. 2002; Kamprath et al. 2009). We have previously shown that CB1 receptor rescue in dorsal telencephalic glutamatergic neurons is not sufficient to restore fear extinction to normal wildtype levels (Ruehle et al. 2013). To assess possible sufficiency of the CB1 receptor expressed in forebrain GABAergic neurons for fear extinction, GABA-CB1-RS mice and their CB1-RS wildtype-like controls and Stop-CB1 knockout-like controls were subjected to cued fear conditioning with a single CS–US pairing of a tone and foot shock, followed by an extinction protocol. Importantly, in a separate batch of animals, no differences in pain sensitivity were observed in response to electric foot shocks of increasing intensity (supplementary Fig. S3a).

**Fig. 9:**
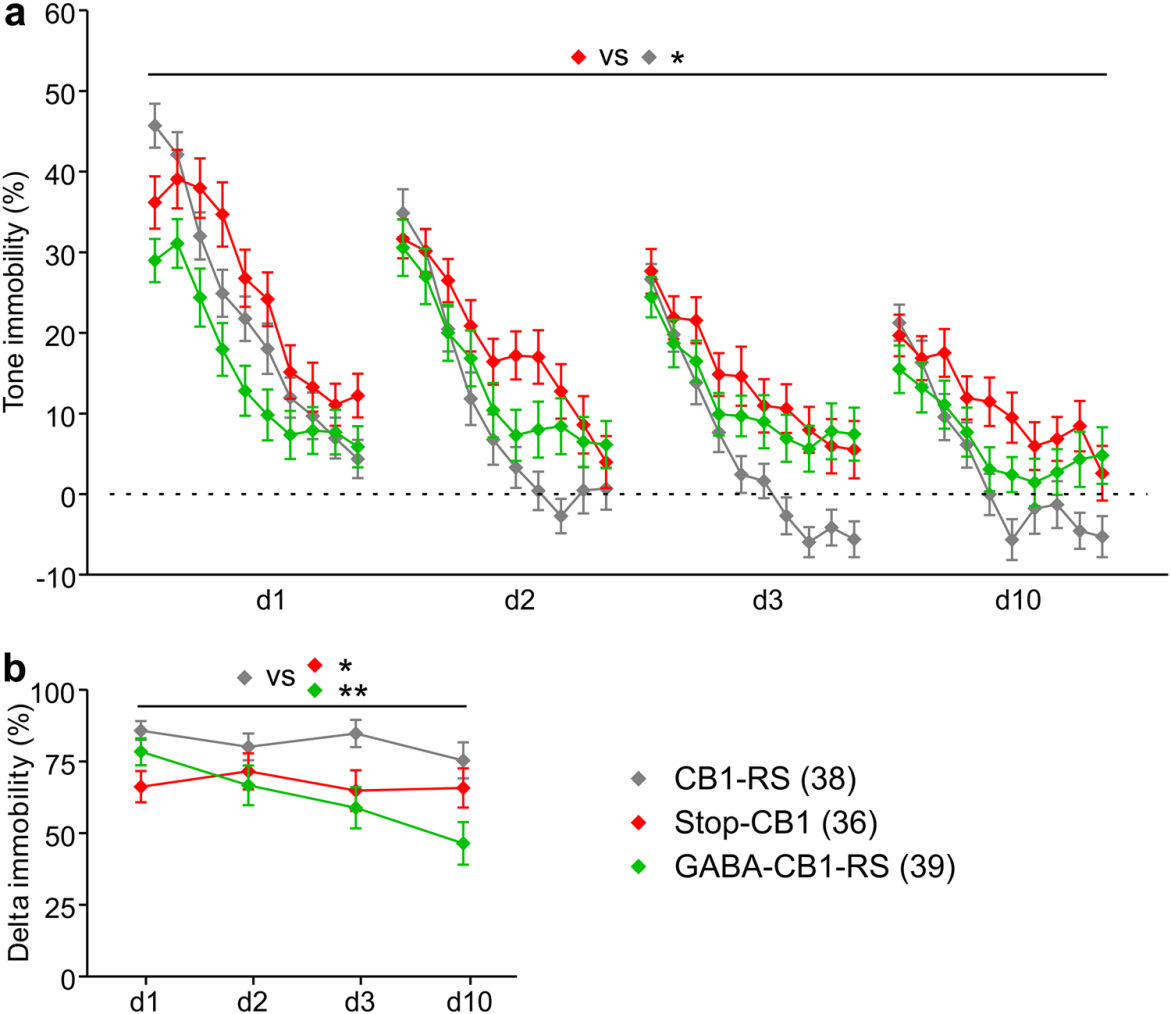
Fear extinction is marginally improved by rescue of the CB1 receptor specifically in forebrain GABAergic neurons. Animals were fear conditioned on day 0 and re-exposed to the tone (CS) for 200 s during extinction sessions on day 1 (*d1*), *d2*, *d3* and *d10* after conditioning. As baseline immobility before the tone differed between groups (see Fig. S4b), subsequent measures of fear extinction were adjusted for baseline immobility using the subtraction method. **a** Immobility to the tone, expressed per 20-s time bin, showed no significant three-way interaction between days, bins, and groups, but a significant effect of group. Post hoc comparisons revealed that Stop-CB1 animals had higher levels of immobility than CB1-RS mice, with intermediate levels in GABA-CB1-RS mice. **b** Within-session fear extinction is expressed as the reduction in immobility between the first and the last 20 s of the 200-s CS presentation, normalized to the initial response (as shown in Fig. S4c) and was significantly stronger in CB1-RS mice than in mice of both other groups. Data are presented as mean ± SEM; animal numbers are indicated; *P < 0.05; **P < 0.01 in repeated measures ANOVA followed by Tukey multiple comparison test; details of statistical analysis in supplementary Table S2

After conditioning on day 0, animals were subjected to extinction sessions on day 1, 2, 3, and 10, and immobility was scored during the 180 s before the onset of the tone, the 200 s of the tone, and the 60 s after the tone (supplementary Fig. S4a). Baseline immobility before the onset of the tone differed between the groups and was significantly reduced in GABA-CB1-RS mice (supplementary Fig. S4b). Therefore, all further responses were adjusted for baseline immobility on the same day by subtracting the average immobility before the tone. The initial fear response to the first CS exposure after conditioning (“immobility first 20 s of CS” minus “baseline immobility”) was lower in GABA-CB1-RS mice than in CB1-RS controls on day 1, but not on further days (supplementary Fig. S4c). Although this might point to a mild deficit in fear retrieval, the same trend was visible in Stop-CB1 animals (*P* = 0.066 vs CB1-RS) in this experiment. In addition, data from a separate batch of animals, where conditioned responses were normalized to those of non-shocked mice for each genotype to obtain another control for the CS-specificity of the response (supplementary Fig. S3b), did not provide support for such a retrieval deficit. As the initial fear response of all groups was similar on subsequent days, between-session extinction seemed to be intact in both Stop-CB1 and GABA-CB1-RS mice. Immobility values over the course of the tone, with baseline immobility subtracted, are shown in Fig. 9a. For all three genotypes, a reduction in immobility was seen over the duration of the 200-s tone given on each extinction day (*P* < 0.001 for time bins). However, there was also a significant interaction of time bin with genotype, implying different extinction between the groups. With no significant three-way interaction between days, bins, and groups, overall immobility to the tone was significantly higher in Stop-CB1 than CB1-RS mice. The total immobility response to the 200-s tone, averaged per day, was significantly higher in Stop-CB1 mice than CB1-RS mice over all days, although there was an interaction with the day (supplementary Fig. S4d). In contrast, immobility after the tone was higher in both Stop-CB1 and GABA-CB1-RS mice than in CB1-RS controls (supplementary Fig. S4e). Within-session extinction (Riebe et al. 2012) was expressed as the reduction in immobility over the 200-s CS presentation normalized to the individual initial response (again, adjusted for baseline immobility). Both Stop-CB1 and GABA-CB1-RS mice showed a deficit in fear extinction over the four days of testing (Fig. 9b). Thus, the immobility response was partly restored in GABA-CB1-RS mice, whereas for within-session extinction no rescue was found. Together, these results point to a limited degree of sufficiency of the CB1 receptor in forebrain GABAergic neurons to mediate appropriate fear extinction.

## Discussion

In the present study, we used a genetic mouse model for conditional CB1 receptor rescue (Ruehle et al. 2013), to investigate whether wildtype-like behaviors could be reconstructed in a CB1-knockout background by restoring expression of the CB1 receptor specifically in forebrain GABAergic neurons. As this rescue mouse model uses the endogenous genomic locus of the CB1 receptor gene, a global (CB1-RS) or conditional rescue (e.g., GABA-CB1-RS) occurs at endogenous sites and induces endogenous expression levels, avoiding ectopic overexpression of the receptor that occurs in other models (Guggenhuber et al. 2010; Naydenov et al. 2014). Using animals without rescue (Stop-CB1, knockout-like) and with global rescue (CB1-RS, wildtype-like) as controls, we assessed the sufficiency for several endocannabinoid-mediated functions of the CB1 receptor subpopulation that is expressed in forebrain GABAergic neurons. Alongside necessity (often investigated using conditional knockout models), sufficiency is the second pillar on the basis of which a causal role can be assigned to a candidate subpopulation.

### Rescue mouse model

Genetic rescue of the CB1 receptor in forebrain GABAergic neurons resulted in the expected patterns of CB1 expression (Katona et al. 1999, 2001; Monory et al. 2006; Steindel et al. 2013) and selectively restored DSI, a form of CB1-mediated short-term synaptic plasticity of inhibitory transmission, without inducing DSE on glutamatergic terminals. The levels of hippocampal CB1 receptor protein and binding in GABA-CB1-RS mice were slightly lower than expected on the basis of previous results obtained in GABA-CB1-KO mice (Steindel et al. 2013). This may be related to differential compensatory effects during development in the conditional knockouts and the conditional rescue mice, or to methodological differences between the studies.

Since the CB1 receptor is mostly located in axon terminals (Kano et al. 2009; Katona and Freund 2012), the location of the protein does not need to match the location of the origin of expression. With many GABAergic neurons being local interneurons, projection areas are close to the soma, and most of the CB1 receptor protein is expected to stay roughly at the site of expression. However, forebrain GABAergic projections have been found interconnecting forebrain regions including the hippocampus, amygdala, and septum (Tóth and Freund 1992; McDonald et al. 2012; Müller et al. 2012; Lübkemann et al. 2015; McDonald and Mott 2017), and also projections from striatum to areas such as the substantia nigra (e.g. da Silva et al. 2015). Some of these GABAergic projections were reported to be positive for cholecystokinin or calbindin (Tóth and Freund 1992; Lübkemann et al. 2015), therefore, might be CB1-positive as well (Marsicano and Lutz 1999; McDonald and Mascagni 2001) and thus are potentially rescued in GABA-CB1-RS mice. Even though it is not a forebrain region, CB1 receptor expression may also be rescued within the substantia nigra itself, as Cre expression was reported here in another mouse line using the Dlx5/6 enhancer (Fu et al. 2012). In general, restoring CB1 receptor function in GABAergic neurons is expected to result in disinhibition (Kano et al. 2009; Katona and Freund 2012). With the lower efficiency of GABAergic CB1 receptors to activate G proteins when compared to those on glutamatergic neurons, at least in the hippocampus (Steindel et al. 2013), smaller behavioral effects might be expected as a consequence of genetic manipulation of CB1 receptor on GABAergic neurons. The necessary roles of subpopulations of the CB1 receptor indeed seem to be more modest for those in GABAergic than in glutamatergic neurons (e.g., Monory et al. 2006, 2007; Bellocchio et al. 2010; Häring et al. 2011; Metna-Laurent et al. 2012; Dubreucq et al. 2012; Llorente-Berzal et al. 2015). Regarding sufficiency, the data so far (this study; Ruehle et al. 2013) seem to suggest less dominance of the contribution of the glutamatergic over that of the GABAergic CB1 subpopulation.

In most, though not all, studies that reported on the necessity of subpopulations of CB1-expressing cells, conditional CB1-knockouts were directly compared only to the wildtype situation (Monory et al. 2006; Azad et al. 2008; Puighermanal et al. 2009; Kamprath et al. 2009; Jacob et al. 2009; Bellocchio et al. 2010; Piet et al. 2011; Metna-Laurent et al. 2012; Rey et al. 2012; Steindel et al. 2013; Fuss et al. 2015; Martín-García et al. 2016). To obtain a more precise assessment of the degree of sufficiency, we included both a wildtype-like (CB1-RS) and a knockout-like (Stop-CB1) control group for CB1 receptor expression (Ruehle et al. 2013). One possible concern of this strategy that should be kept in mind is that due to the nature of the mouse lines used; all CB1-RS mice were raised by two wildtype-like parents, whereas in both other groups (Stop-CB1 and GABA-CB1-RS) both parents were at least mostly deficient for CB1. Therefore, aside from the developmental differences of presence or lack of the CB1 receptor in the offspring themselves, there might be some unavoidable differences due to pre- and postnatal parental effects of CB1 (Fride 2008; Sun and Dey 2012; Maccarrone et al. 2014; de Salas-Quiroga et al. 2015). To minimize differences emerging between the mouse lines due to genetic drift, they are regularly interbred. To further avoid this possible confounding influence, all genotypes needed for experiments (CB1-RS, Stop-CB1 and GABA-CB1-RS) could be combined in one breeding strategy. However, because of the large number of animals with unusable genotypes resulting from such a mating system, this strategy is unfeasible and unethical.

### Susceptibility to different types of seizures differentially regulated by CB1

A previous report detected no necessary role for GABAergic CB1 in the protection against kainic acid-induced seizures (Monory et al. 2006). Therefore, to identify possible differences between two groups with expected high seizure susceptibility (Stop-CB1 and GABA-CB1-RS), a relatively low dose of kainic acid was administered. At the first observation time-point, only Stop-CB1 mice had significantly higher scores than CB1-RS control mice, but apart from this partial sufficiency in the initial response, no sufficiency could be detected for the CB1 receptor in forebrain GABAergic neurons. Nevertheless, this absence of an important role for GABAergic CB1 receptors in the susceptibility to epileptiform seizures may be specific to the model of seizures induced by kainic acid. Since GABAergic signaling is generally protective against seizures (but see Snodgrass 1992), the overall expectation would be that CB1-mediated suppression of GABA transmission should lead to stronger epileptiform activity (Alger 2006). However, this prediction is complicated by the specific actions of kainic acid (Carta et al. 2014; Soltesz et al. 2015). The direction of the effect of kainic acid on GABA release seems to be dose-dependent, with low doses potentiating and high doses depressing inhibitory transmission (Jiang et al. 2001; Prager et al. 2016). Additionally, kainate receptor agonists may activate GABAergic neurons that do not express the CB1 receptor, whereas it may inhibit those that are CB1-positive (Lourenço et al. 2010, 2011; Daw et al. 2010; Carta et al. 2014). Furthermore, it has been shown that the ability of CB1 receptor signaling to reduce GABAergic transmission is strongly reduced or even abolished at high firing rates of these neurons (Földy et al. 2006). Together, these characteristics complicate the prediction whether GABAergic CB1 receptors should improve or aggravate these excitotoxic seizures. Recently, the more intuitive seizure-exacerbating effect of GABAergic CB1 receptors was reported for the kindling model of temporal lobe epilepsy, with the conditional knockouts showing seizures of shorter duration (von Rüden et al. 2015). In contrast, the GABA-CB1-RS mice rather seem to point towards a protective role of GABAergic CB1 receptor for susceptibility to different types of seizures. GABA-CB1-RS mice, similar to Glu-CB1-RS mice and CB1-RS controls, required a higher dose of pentylenetetrazole and exhibited a longer latency to seizure than Stop-CB1 mice (de Salas-Quiroga et al. 2015). In addition, GABAergic CB1 receptors may have attenuated the initial, if not the ultimate, reaction in the kainic acid model. Moreover, in our experimental animals, the mortality due to spontaneous seizures observed in the Stop-CB1 group was absent in GABA-CB1-RS mice. Therefore, the exact role of GABAergic CB1 receptor in seizure susceptibility may vary between these different types of seizures.

### Innate anxiety: redundancy of anxiolytic CB1-mediated effects

In the EPM and LD tests, we found a large degree of sufficiency of the CB1 receptor in forebrain GABAergic neurons for appropriate anxiety-like behavior; in GABA-CB1-RS mice, the increase in anxiety-like behavior observed in Stop-CB1 mice was reversed. This may seem counterintuitive. After all, GABAergic CB1 receptors have been shown to be necessary for the anxiogenic effects of high-dose cannabinoid treatment (Rey et al. 2012). Furthermore, benzodiazepines, which promote inhibitory neurotransmission by positive allosteric modulation of GABAA receptors, are widely used for their anxiolytic properties (Griebel and Holmes 2013), whereas GABAergic CB1 receptor should decrease GABAergic signaling. At first glance, the anxiolytic role of GABAergic CB1 receptor also seems to be in contradiction with the (partial) sufficiency we previously reported for the glutamatergic CB1 receptor (Ruehle et al. 2013). The CB1 receptor in glutamatergic neurons reduces excitatory neurotransmission and seems to be responsible for the overall network activity-reducing effect of (endo)cannabinoids (Azad et al. 2003; Piet et al. 2011). Thus, glutamatergic CB1 receptor rescue may well reduce BLA hyperactivity and hyperexcitability, features that are associated with excessive anxiety (Prager et al. 2016), whereas GABAergic CB1 receptor rescue could be expected to have the opposite effect. However, it is important to realize that GABA-CB1-RS mice exhibit reduced GABAergic signaling only in specific forebrain synapses. Many brain regions play a role in anxiety (Tovote et al. 2015); regions containing GABAergic interneurons, regions containing GABAergic projection neurons, some of which do and some of which do not express CB1 in the wildtype situation. In the GABA-CB1-RS mouse, the CB1 receptor is rescued in a specific subpopulation of these inhibitory connections, and GABAergic CB1 rescue can thus not be expected to result in a general reduction in GABA signaling. More specifically, the large majority of GABAergic CB1-containing neurons is cholecystokininergic (Marsicano and Lutz 1999; McDonald and Mascagni 2001; Katona et al. 2001). Cholecystokinin has well-known anxiogenic effects, at least in part via the amygdala and prefrontal cortex (Bowers et al. 2012; Li et al. 2013; Vialou et al. 2014), and thus restored CB1 receptor expression in these neurons may reduce anxiety-like behavior via a reduction of cholecystokinin release. This is in accordance with an anxiolytic effect of stimulation of the CB1 receptor in the prefrontal cortex with low doses of cannabinoids (Rubino et al. 2008a, b). Furthermore, a rescue of CB1 receptors on inhibitory projections that promote anxiety, such as those from the CeA to the bed nucleus of the stria terminalis (BNST) or from the lateral septum to the hypothalamus (Tovote et al. 2015), could be expected to reduce anxiety. An anxiolytic role has also recently been described for GABAergic CB1 receptor in a running paradigm (Fuss et al. 2015). Therefore, although CB1 receptors in glutamatergic and GABAergic neurons both provide a degree of sufficiency for appropriate levels of innate anxiety, they probably do so through distinct mechanisms.

The anxiogenic effect resulting from CB1 receptor deficiency has been described to depend strongly on the aversiveness of the test and its conditions (Haller et al. 2004). Accordingly, several studies have reported unaltered anxiety-like behavior in CB1 null-mutants and conditional knockouts (Jacob et al. 2009; Dubreucq et al. 2012; Rey et al. 2012). However, there are some indications for a necessary role for GABAergic CB1 receptors in anxiety-like behavior in the LD test, as discussed by Dubreucq and colleagues (2012). With this limited necessity reported for GABAergic CB1 receptor, the large degree of sufficiency we found for this subpopulation of the receptor is unexpected and remarkable.

### Extinction of learned fear: no substantial sufficiency of GABAergic CB1 receptors

Whereas fear acquisition and initial fear expression after conditioning are generally not affected in CB1 receptor null mutants or Stop-CB1 mice, fear extinction is impaired (Marsicano et al. 2002; Kamprath et al. 2009; Ruehle et al. 2013). This impairment was not restored by a rescue of the CB1 receptor in dorsal telencephalic glutamatergic neurons (Ruehle et al. 2013). Here, we found a slight reduction of fear responses after a GABAergic rescue of the CB1 receptor, and hence limited sufficiency of this CB1 subpopulation.

GABA-CB1-RS mice showed less immobility in the pre-tone period of the extinction sessions. It may, therefore, be suggested that these animals display increased overall locomotor activity. However, we did not detect changes in the distance traveled in the open field by GABA-CB1-RS mice, and their activity rather tended to be reduced in the EPM test, with a significantly lower distance traveled than Stop-CB1 mice but no difference in total arm entries to either of the control groups. In GABA-CB1-KO mice, both increased and decreased activity have been reported as well (Häring et al. 2011; Dubreucq et al. 2013; Fuss et al. 2015). The influence of GABAergic CB1 receptor on locomotor activity thus seems to be variable, depending on the paradigm and exact circumstances. Regardless of whether the reduced baseline immobility correlates with overall activity and whether this is specifically regulated by GABAergic CB1 receptors, for the evaluation of fear responses, immobility during the tone was normalized to that during the pre-tone to exclude possible confounding for the interpretation of fear extinction.

In the basal amygdala, contextual fear extinction increases CB1 receptor protein on GABAergic terminals around active (but not silent) fear neurons and possibly around extinction neurons (Trouche et al. 2013). This would be expected to dampen inhibition of both, oppositely acting, neuronal populations, the net result of which might affect fear extinction only moderately. It is, therefore, not surprising that the behavioral consequences observed in GABA-CB1-KO mice were mild and rather variable (Metna-Laurent et al. 2012; Dubreucq et al. 2012; Llorente-Berzal et al. 2015). Obviously, the BLA is only one of several regions that are involved in fear regulation and contain CB1 receptors on GABAergic terminals. Other major nodes in the fear and extinction network such as the hippocampus and prefrontal cortex (Tovote et al. 2015) also express the CB1 receptor in GABAergic neurons (Lutz et al. 2015). GABA-CB1-RS mice also showed substantial CB1 receptor agonist binding in these regions (Figs. 1, 2). Since the prelimbic and infralimbic portions of the prefrontal cortex have opposite effects on fear responses during extinction training (Vidal-Gonzalez et al. 2006; Sierra-Mercado et al. 2011), the GABAergic CB1 receptors in these two regions might again counteract each other’s actions, causing only mild changes in fear responses. However, much of the precise connectivity and exact function in fear extinction between these regions remains to be characterized (Tovote et al. 2015), and complex interactions are emerging, with a circuit of different types of prefrontal interneurons determining fear expression (Courtin et al. 2014).

## Conclusions

In this study, we used a Cre-mediated cell-type-specific CB1 receptor rescue strategy in a CB1-null background to attempt to restore normal behaviors by rescuing the receptor to its endogenous levels solely in forebrain GABAergic neurons. This subpopulation of the CB1 receptor was found to convey no substantial protection against kainic acid-induced epileptiform seizures, but abolished the spontaneous seizures observed in mice lacking the CB1 receptor. We showed differential sufficiency of the GABAergic CB1 subpopulation in two distinct paradigms of emotional behavior. A rescue of GABAergic CB1 receptors only slightly improved the fear extinction deficit found in null-mutants, whereas innate anxiety-like behavior was largely restored to normal. This illustrates that innate and learned fear and anxiety are separate phenomena that are regulated by the endocannabinoid system through distinct mechanisms. Together with previous results from the rescue in glutamatergic neurons it also indicates that there is at least partial redundancy between CB1 receptors in different cell types for appropriate innate anxiety, whereas for proper fear extinction, a more intact and “balanced” CB1-mediated feedback may be required. Here, we have defined the degree of sufficiency of the forebrain GABAergic subpopulation of the CB1 receptor for several different outputs of the endocannabinoid system. With these new insights into its sufficiency, this is an essential addition to previous studies on the necessity of this subpopulation. Only when both conditions are fulfilled can a real causal role be determined conclusively. Comparing the present results with those previously obtained with conditional CB1-deficiency, it is also evident that necessity and sufficiency do not always match. Furthermore, the basic characterization of these mice with GABAergic (this study) and glutamatergic (Ruehle et al. 2013) rescue of the CB1 receptor paves the way for further studies in these mouse models. The CB1 rescue mouse is also a useful model to study the sufficiency of CB1 subpopulations for pharmacological and biochemical properties. Issues that remain to be clarified include the relative sufficiency for the low-dose and high-dose cannabinoid effects on anxiety (analog to Rey et al. 2012), and the efficiency of G protein coupling of the CB1 receptor in different cell types (analog to Steindel et al. 2013), in the hippocampus and other brain regions or tissues.

## Electronic supplementary material

Below is the link to the electronic supplementary material.

Supplementary material 1 (DOCX 4502 KB)
