# Supplementary material for: Addressing sufficiency of the CB1 receptor for endocannabinoid-mediated functions through conditional genetic rescue in forebrain GABAergic neurons
Source: Brain Struct Funct. 2017 Apr 9;222(8):3431–52. doi: 10.1007/s00429-017-1411-5 (PMC5676814; doi:10.1007/s00429-017-1411-5)
Supplement: Supplementary file 1 — Supplementary material 1 (DOCX 4502 KB) [file 429_2017_1411_MOESM1_ESM.docx]

**Addressing sufficiency of the CB1 receptor for endocannabinoid-mediated functions through conditional genetic rescue in forebrain GABAergic neurons**

Floortje Remmers, Maren D Lange, Martina Hamann, Sabine Ruehle, Hans-Christian Pape, Beat Lutz

Corresponding author: Floortje Remmers, [remmersf@uni-mainz.de](mailto:remmersf@uni-mainz.de).

Institute of Physiological Chemistry, University Medical Center of the Johannes Gutenberg University Mainz, 55128 Mainz, Germany

Supplementary Figures


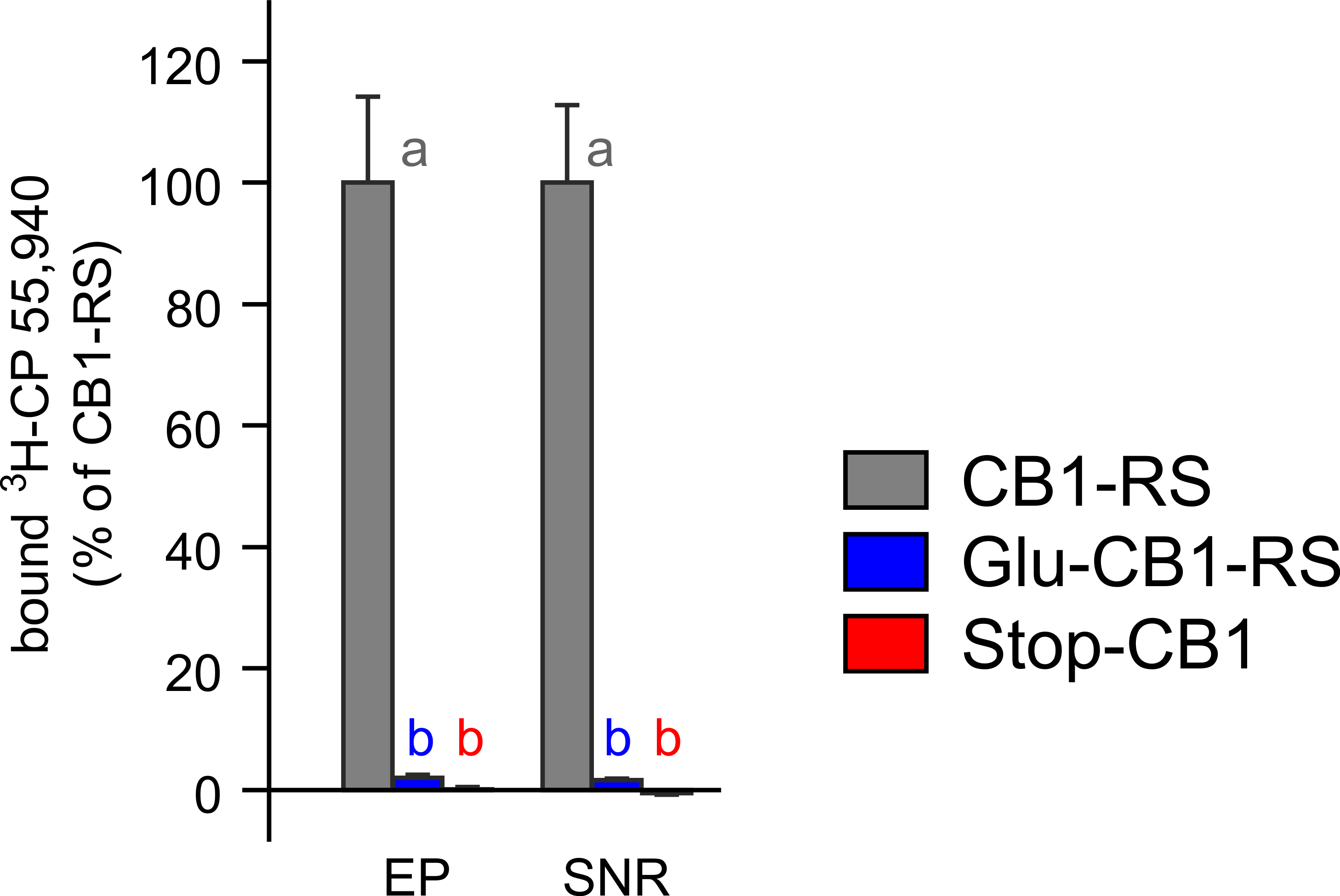


Fig. S1 Additional quantification of Glu-CB1-RS autoradiography. Quantification of CB1 receptor ligand binding intensity of 2 regions from autoradiograms that were published by Ruehle et al in (2013) but not quantified at the time: EP, entopeduncular nucleus (mean of 7 sections/animal); SNR, substantia nigra (mean of 6 sections/animal). Signal intensity was quantified per brain region, relative to that in CB1-RS mice (n=3 brains per group) in autoradiograms of coronal sections of CB1-RS, Glu-CB1-RS, and Stop-CB1 brains. Values of columns labeled with the same letter (a, b, or c) are not significantly different from each other in repeated measures ANOVA followed by Tukey multiple comparison test; data are expressed as mean + SEM; details of statistical analysis in supplementary Table S2


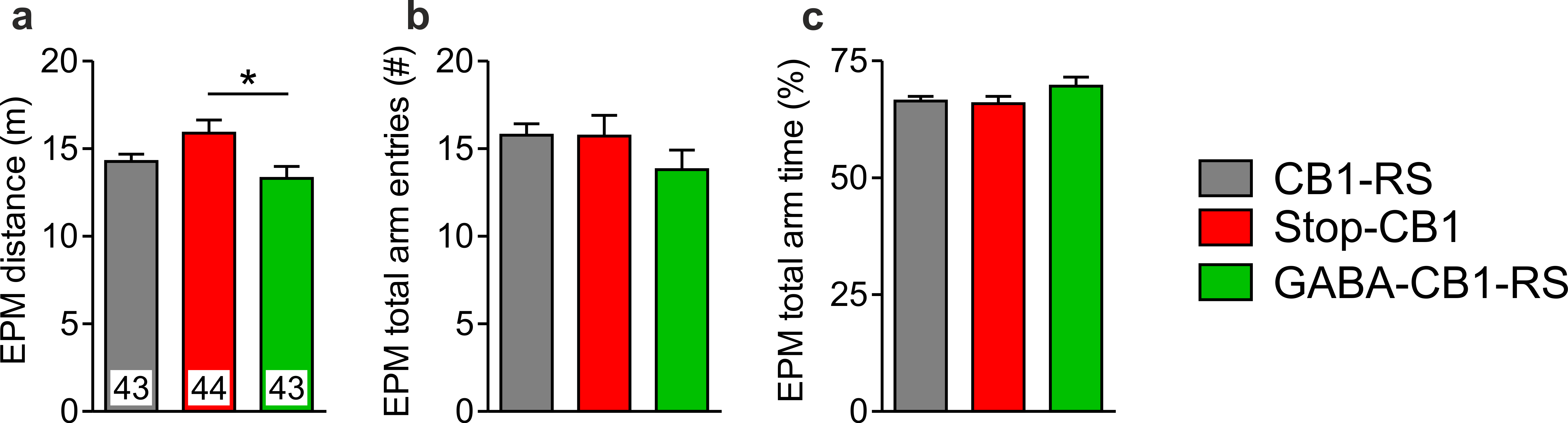


Fig. S2 Locomotion in the elevated plus-maze test. In the elevated plus-maze (EPM) test, (a) the Stop-CB1 mice covered significantly more distance than GABA-CB1-RS mice, but both did not differ from CB1-RS mice. (b) The total number (#) of entries into all arms (open and closed), another proxy for total locomotion in the EPM test, did not differ between the groups. (c) All groups spent similar proportions of the 5-min test time in the arms and center of the EPM. Data are expressed as mean + SEM; animal numbers are indicated in the graphs; *, *P* < 0.05 in one-way ANOVA followed by Tukey multiple comparison test; details of statistical analysis in supplementary Table S2


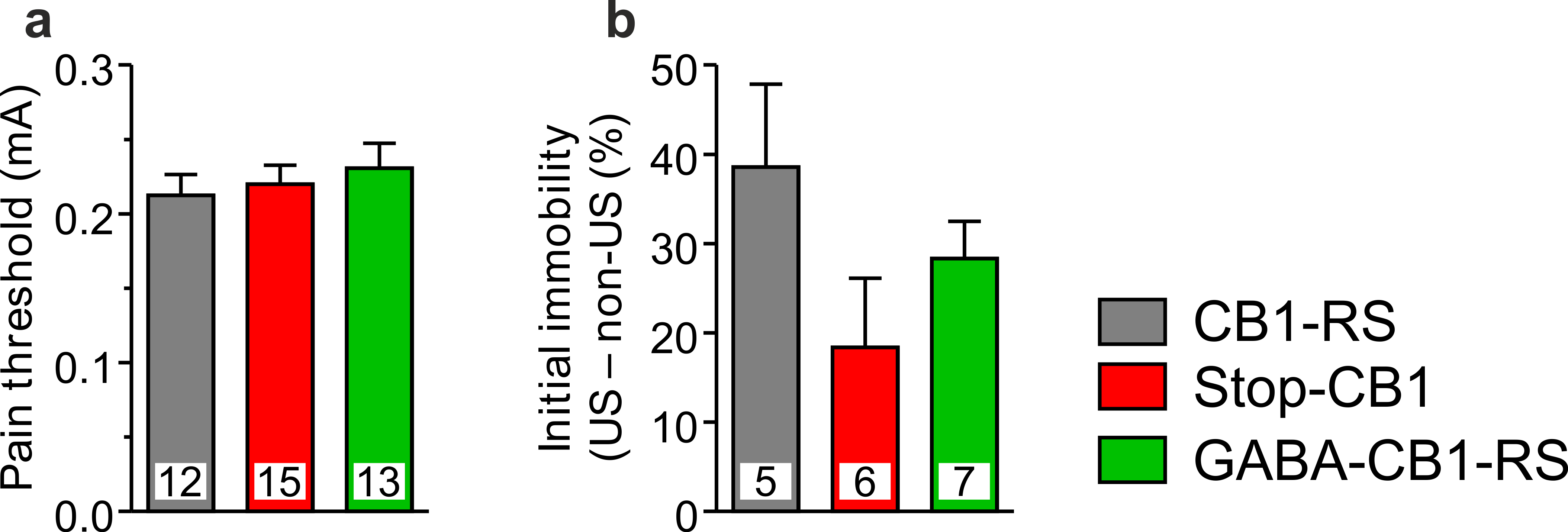


Fig. S3 Similar pain threshold and fear expression between groups. (a) Pain threshold was determined in a separate batch not subjected to fear conditioning. No significant differences were observed between CB1-RS, Stop-CB1, and GABA-CB1-RS mice. (b) Furthermore, no significant differences were detected between CB1-RS, Stop-CB1, and GABA-CB1-RS mice in the initial fear response upon the first re-exposure to the tone after conditioning when this response was adjusted for the response to the tone of animals of the same genotype that had not received a foot-shock the previous day. Data are expressed as mean + SEM; animal numbers are indicated in the graphs. Data were analyzed by one-way ANOVA; details of statistical analysis in supplementary Table S2


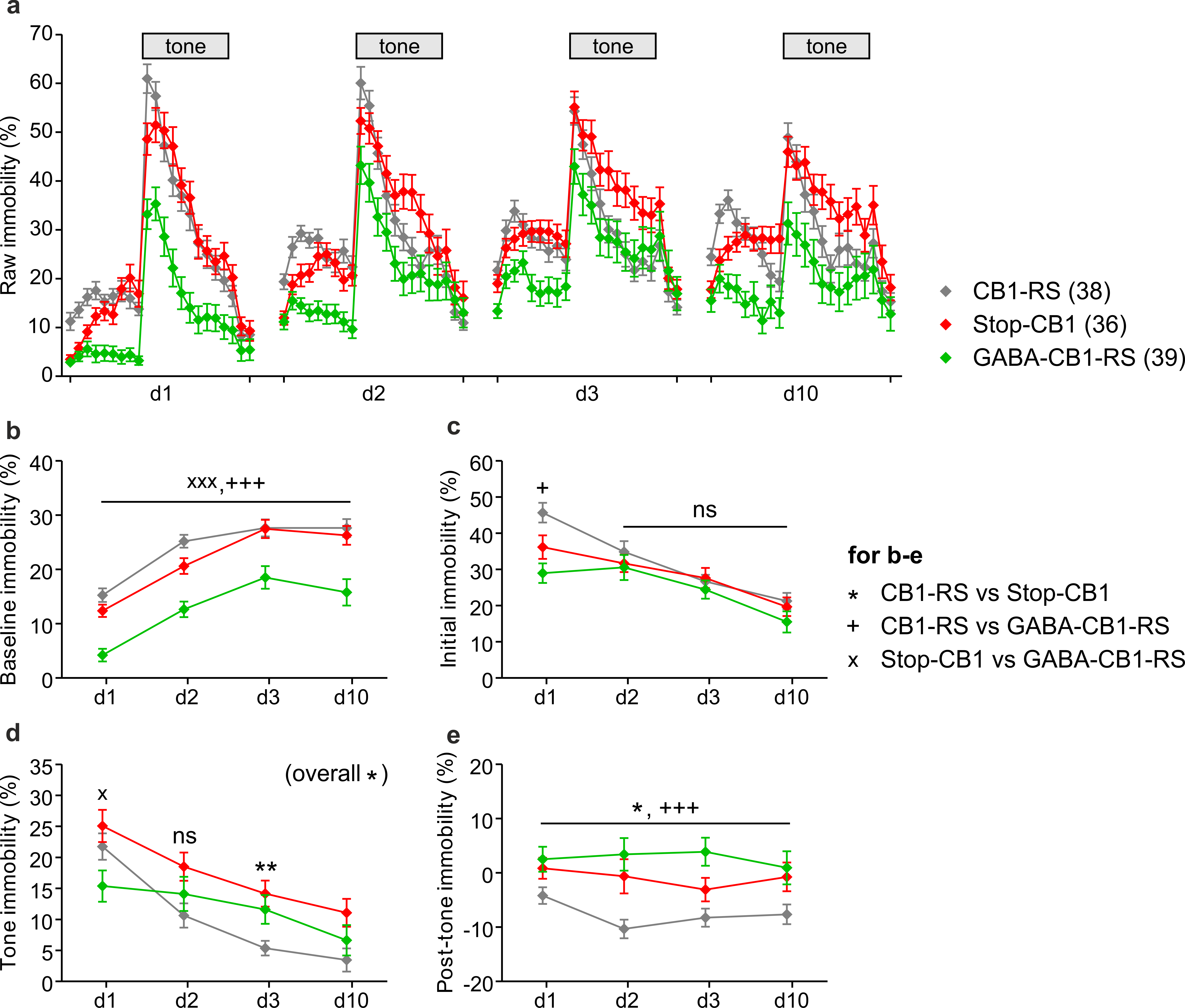


Fig. S4 Immobility after fear conditioning. Animals were fear conditioned on day 0 (d0) and re-exposed to the tone (CS) for 200 seconds during extinction sessions on d1, 2, 3 and 10 after conditioning. (a) Unadjusted immobility throughout the extinction trials in 20-second time bins. (b) Baseline immobility in the 180 seconds before the onset of the CS was lower in GABA-CB1-RS mice than in their wildtype-like (CB1-RS) and knockout (Stop-CB1) controls. Because of these differences, subsequent measures of fear extinction were adjusted for baseline immobility using the subtraction method. (c) The initial fear response (*adjusted for baseline immobility*) during the first 20 seconds of the 200-second presentation of the CS was lower in GABA-CB1-RS mice than in CB1-RS mice on the first day of extinction only, with Stop-CB1 mice showing intermediate values (*P* = 0.066 vs CB1-RS). Since in Fig. 9a there was a significant interaction between day and group, (d) the total immobility to the tone, averaged per day, shows different group effects for the different days, although generally Stop-CB1 mice showed higher immobility during the tone while CB1-RS mice exhibited lower levels and immobility of GABA-CB1-RS mice was mostly intermediate (overall * Stop-CB1 vs CB1-RS for all days together, d10 *P* = 0.051 for Stop-CB1 vs CB1-RS). (e) Immobility after the tone was elevated in both Stop-CB1 and GABA-CB1-RS mice. Data are presented as mean ± SEM; animal numbers are indicated. Data in b-e were analyzed by repeated measures ANOVA followed by Tukey multiple comparison test, or (if there was a significant interaction between day and group) per day by post-hoc simple effect analysis with Sidak correction and are indicated by * between CB1-RS and Stop-CB1, + between CB1-RS and GABA-CB1-RS, x between Stop-CB1 and GABA-CB1-RS (1, *P* < 0.05; 2, *P* < 0.01; 3, *P* < 0.001; ns, not significant); details of statistical analysis in supplementary Table S2

Supplementary Tables

Table S1 Genotyping primers

| **primer** | **sequence 5'-3'** | **band sizes (bp)** | |
| --- | --- | --- | --- |
| Stop-CB1 fwd1 | CAAGAAATGAGAACCGTGTC | wt | 543 |
| Stop-CB1 fwd2 | TGTGTGAATCGATAGTACTAAC | Stop | 462 |
| Stop-CB1 rev | GTTCTCCTTGAACGATGAGA | RS | 577 |
| DLX-Cre fwd | GGCTCAAGACTCGTCAAAATC | wt | … |
| DLX-Cre rev | CGCGCCTGAAGATATAGAAGA | Cre | 350 |
| EIIa-Cre fwd | CGGCATGGTGCAAGTTGAATA | wt | … |
| EIIa-Cre rev | GCGATCGCTATTTTCCATGAG | Cre | 300 |

bp, base pairs; DLX-Cre, specific primers for DLX-Cre; EIIa-Cre, general Cre primers; fwd, forward primer; RS, rescued; rev, reverse primer; wt, wildtype.

Table S2 Details of statistical analyses. Animal numbers and cell/animal numbers (n) are given for CB1-RS, Stop-CB1 and GABA-CB1-RS. AON, anterior olfactory nucleus; B, bin; BLA, basolateral amygdala; CA1, cornu ammunis 1 region of hippocampus; CeA, central amygdala; co, covariate; CPu, caudate putamen; D, day; dist, distance; DSE, depolarization-induced suppression of excitation; DSI, depolarization-induced suppression of inhibition; EP, entopeduncular nucleus; EPM, elevated plus-maze; FC, fear conditioning; G, genotype; GP, globus pallidus; Hip, hippocampus; HT, hypothalamus; KA, kainic acid induced seizures; LD, light/dark test; MGN, medial geniculate thalamic nucleus; ns, not significant; OF, open field; R, region; RM, repeated measures; SNR, substantia nigra; T, time

Table S2, continued

Table S2, continued

Table S2, continued
